# Identification of GXXXXG motif in Chrysophsin-1 and its implication in the design of analogs with cell-selective antimicrobial and anti-endotoxin activities

**DOI:** 10.1038/s41598-017-03576-1

**Published:** 2017-06-13

**Authors:** Amit Kumar Tripathi, Tripti Kumari, Munesh Kumar Harioudh, Pranjal Kumar Yadav, Manoj Kathuria, P. K. Shukla, Kalyan Mitra, Jimut Kanti Ghosh

**Affiliations:** 1 Molecular and Structural Biology Division, CSIR-Central Drug Research Institute, Sector 10, Jankipuram Extension, Sitapur Road, Lucknow, 226 031 India; 2 Microbiology Division, CSIR-Central Drug Research Institute, Sector 10, Jankipuram Extension, Sitapur Road, Lucknow, 226 031 India; 3 Electron Microscopy Unit, CSIR-Central Drug Research Institute, Sector 10, Jankipuram Extension, Sitapur Road, Lucknow, 226 031 India

## Abstract

Marine fish antimicrobial peptide, chrysophsin-1 possesses versatile biological activities but its non-selective nature restricts its therapeutic possibilities. Often small alterations in structural motifs result in significant changes in the properties of concerned proteins/peptides. We have identified GXXXXG motif in chrysophsin-1. Glycine residue(s) of this motif in Chrysophsin-1 was/were replaced with alanine, valine and proline residue(s). Of these, proline-substituted Chrysophsin-1 analogs exhibited significantly reduced cytotoxicity towards mammalian cells. Further, these analogs showed broad-spectrum activity against Gram-positive, Gram-negative bacteria, Methicillin-resistant *Staphylococcus aureus* strains and fungi and also retained antibacterial activity in presence of physiological salts, serum and at elevated temperatures indicative of their therapeutic potential. These Chrysophsin-1 analogs also inhibited lipopolysaccharide (LPS) induced pro-inflammatory responses in THP-1 cells and in murine primary macrophages. One of these single proline-substituted Chrysophsin-1 analogs inhibited LPS-stimulated pro-inflammatory cytokine production in BALB/c mice and elicited appreciable survival of mice administered with a lethal dose of LPS in a model of severe sepsis. The data for the first time showed the implication of GXXXXG motifs in functional and biological properties of an antimicrobial peptide and could be useful to design novel anti-microbial and anti-endotoxin peptides by employing this motif.

## Introduction

The clinical efficacies of most antibiotics have declined drastically as a result of the emergence of dominant resistance in microorganisms against them^1^. It seems that the indiscriminate use of antibiotics put an evolutionary pressure on pathogens to develop strategies for nullifying their effects^2^. This bacterial resistance has given an impetus to the research in a family of peptides known as antimicrobial peptides (AMPs) or host defense peptides (HDPs) that possess significant antimicrobial and immunomodulatory activities^3–5^.

Marine fishes have been proved to be a rich source of antimicrobial peptides^6^. Chrysophsins (Chrysophsin-1,-2 and-3) were first isolated as amphipathic, α-helical antimicrobial peptides from the gill cells of red sea bream (*Chrysophrys major*) (Table 1)^7^. Despite displaying various biological activities such as antibacterial, anti-endotoxic and antitumor activities, chrysophsins are highly non-cell-selective and kill the bacterial and mammalian cells with equal efficacy. A Chrysophsin-1 analog with deletion of its -RRRH from its C-terminus showed reduced cytotoxicity against human lung fibroblasts^8^. Though antimicrobial activity of this Chrysophsin-1 deletion analog has not been reported, presented biophysical data indicated that the deletion of the motif could reduce its activity against microorganisms also. Thus the design of non-toxic analogs of Chrysophsin-1 without compromising its antimicrobial properties remain a challenging issue.

**Table 1:** Amino Acid Sequences of three different isoforms of Chrysophsin; Glycine residues of the identified GXXXXG motifs are marked in bold and underlined.

Peptide	Sequence	Length	Molecular Weight	Znet	%Hydrophobicity
Chrysophsin-1	**G**XXXX**G**XXXX**G**XXXX**G**FF**G**WLIK**G**AIHA**G**KAIH**G**LIHRRRH	25	2892.79	+5	48%
Chrysophsin-2	FF**G**WLIR**G**AIHA**G**KAIH**G**LIHRRRH	25	2920.80	+5	48%
Chrysophsin-3	FIGLLISAGKAIHDLIRRRH	20	2286.97	+4	50%

The structural motifs present in AMPs also play crucial roles in the biological activity of their parent peptides. Small alterations in these motifs result in significant changes in the properties of concerned proteins/peptides. We identified a combination of two glycine residues with four amino acid residues in between them in Chrysophsin-1 what we call as ‘GXXXXG’ i.e. there is a recurrence of glycine after every four amino acids throughout the sequence of Chrysophsin-1 which was not reported before in this peptide. Interestingly this motif is also present in Chrysophsin-2, the second isoform of Chrysophsin which is also equally cytotoxic. Therefore, realizing the plausible implication of this motif in the biological activity of Chrysophsin-1, we set to unravel the importance of this motif in cytotoxicity, anti-bacterial and anti-endotoxic activities of this AMP by selectively replacing the glycine residue(s) of these motifs with proline, alanine and valine residue(s).

## Results

### Design of Chrysophsin-1 variants

Chrysophsin-1 is a 25-residue peptide and has a strong sequence homology with another isoform, Chrysophsin-2 which differs only at the seventh position where arginine is present instead of lysine (Table 1). The positive charges in Chrysophsin-1 are localized at the C-terminus mainly due to the presence of RRRH whereas the N-terminus is hydrophobic due to FFGWLI segment. The GXXXXG motif is distributed throughout the Chrysophsin-1. Since glycine to proline substitutions do not change the physiochemical properties of the peptide, proline substitutions were preferred for looking into the role of GXXXXG motif in Chrysophsin-1 (Table 2). The HPLC profiles for purifications of these peptides are shown (Figure S1, Supplementary Information). However, it is to be mentioned that proline and glycine show contrasting conformational restrictions while the former possesses the highest restriction, the latter amino acid displays the least restrictions^9^. Interestingly, yet proline has been found with glycine in transmembrane domains of several ion channel proteins as well as in a number of membrane active peptides with ion channel activity^10, 11^. Thus considering all the literature, it was of interest to look into the effects of glycine to proline substitutions in GXXXXG motifs of chrysophsin-1.

**Table 2:** Chrysophsin-1 (Chr-1) and its designed novel analogs with glycine residue(s) of GXXXXG motifs substituted with proline residue(s).

S.No	Peptide	Sequence	Charge (Z_net_)	Calculated M.W.	Observed M.W.	% Hydrophobicity
1	Chr-1	**G**XXXX**G**XXXX**G**XXXX**G**FF**G**WLIK**G**AIHA**G**KAIH**G**LIHRRRH	+5	2892.79	2892.81	48%
2	G8P-Chr-1	FF**G**WLIK***P***AIHA**G**KAIH**G**LIHRRRH	+5	2932.85	2932.88	48%
3	G13P-Chr-1	FF**G**WLIK**G**AIHA***P***KAIH**G**LIHRRRH	+5	2932.85	2932.86	48%
4	G18P-Chr-1	FF**G**WLIK**G**AIHA**G**KAIH***P***LIHRRRH	+5	2932.85	2932.86	48%
5	G13,18P-Chr-1	FF**G**WLIK**G**AIHA***P***KAIH***P***LIHRRRH	+5	2972.91	2972.94	48%

Glycine residues of GXXXXG motifs are marked as bold and underlined letters whereas proline residues are marked in bold, underlined and italics.

Alanine and valine substitutions were also made in place of these glycine residues (Table S1, Supplementary Information). However, the substitutions with single and double alanine or valine residue(s) in GXXXXG motifs did not lead to any significant reduction of cytolytic activity of Chrysophsin-1 against human red blood cells (hRBCs) and therefore further studies with these analogs were abandoned (Figure S2, Supplementary Information).

### Substitution of a single glycine residue with a proline residue in GXXXXG motif reduced cytotoxicity of Chrysophsin-1

**Figure 1:**
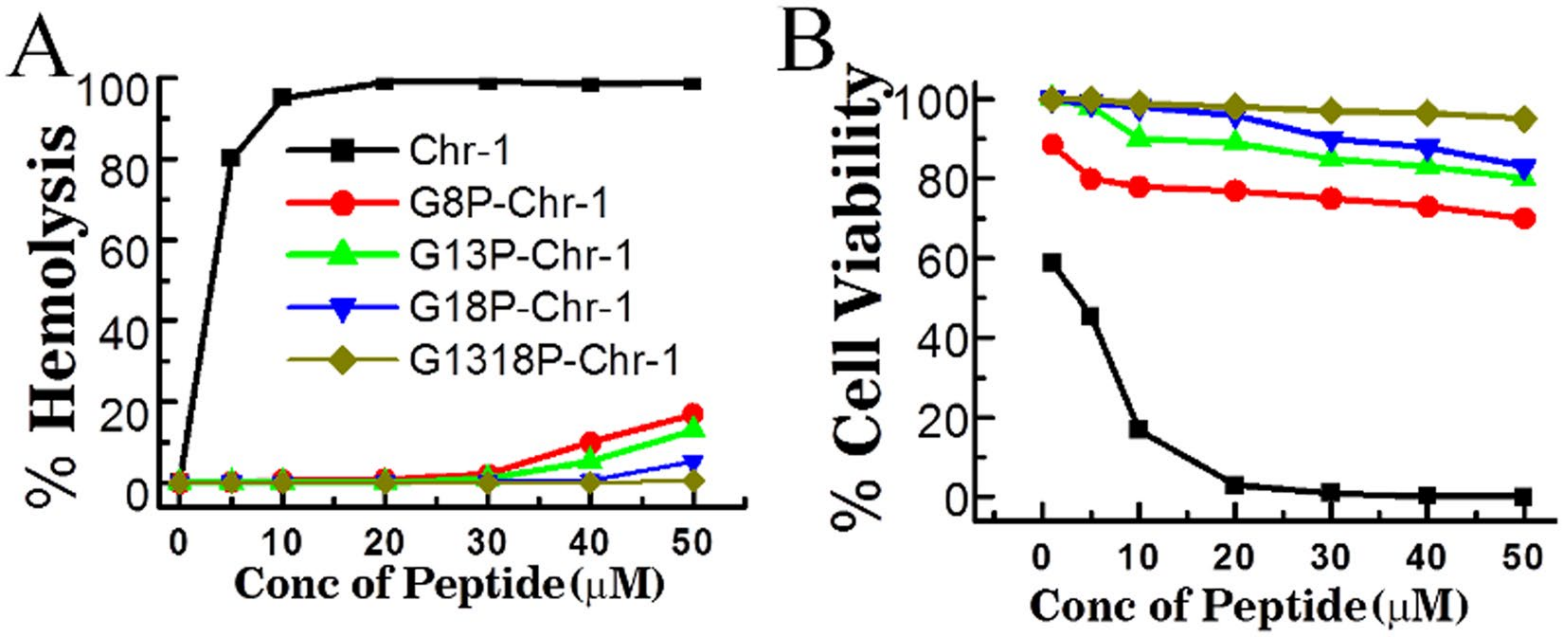
Cytotoxicity of Chr-1 and its analogs. (**A**) and (**B**) Shows dose-dependent cytotoxicity of Chrysophsin-1 and its variants (hemolytic activity) and NIH-3T3 cells (MTT assay for cell viability) respectively.

Despite significant antibacterial, anti-endotoxin and antitumor activities, lack of cell selectivity diminished the utility of Chrysophsin-1 as a peptide antibiotic. However, we observed a significant effect of replacement of glycine residue(s) with proline residue(s) within the identified GXXXXG motif on the cytotoxicity of Chrysophsin-1. The first analog, G8P-Chr-1 in which the glycine residue at the 8^th^ position was substituted with a proline residue showed a marked reduction in cytolytic activity against human red blood cells (hRBCs) (Fig. 1A). Similarly, the other two single glycine-substituted chrysophsin-1 analogs viz. G13P-Chr-1 and G18P-Chr-1 showed drastically reduced hemolytic activity against hRBCs. The double proline-substituted chrysophsin-1-analog, G13,18P-Chr-1 in which two glycine residues of GXXXXG motifs at 13^th^ and 18^th^ positions were replaced with two proline residues showed an even further reduction in hemolytic activity and was practically non-hemolytic up to the maximum tested peptide concentration (50 µM). A similar trend was observed in MTT assays, performed with NIH-3T3 cells in presence of these peptides (Fig. 1B).

### Chrysophsin-1 variants showed antibacterial activity comparable to that of the native peptide

Anti-microbial activity of Chrysophsin-1 and its analogs were examined against various Gram-positive, Gram-negative bacteria and fungi. The proline-substituted chrysophsin-1 analogs appreciably retained the activity of the native peptide against both the Gram-positive and Gram-negative bacteria. The Minimum inhibitory concentration (MIC) of chrysophsin-1 and its non-toxic variants against MRSA and Multi-Drug Resistant (MDR) strains of *S. aureus* were in the range of 1.6–3 μM (Table 3). The assay of antimicrobial activity of Chrysophsin-1 and its analogs was also extended to fungal strains. Except for the double proline-substituted Chrysophsin-1-analog i.e. G13,18P-Chr-1, all the other four analogs showed appreciable antifungal activity against *Candida albicans* (ATCC 10231), *Cryptococcus neoformans*, *Candida parapsilosis* (ATCC 22019) and patient isolates of *Sporothrix schenckii*.

**Table 3:** Antibacterial and antifungal activity of Chrysophsin-1 and its variants.

Minimum inhibitory concentration (MIC) in µM							
Gram-Negative Bacteria	Chr-1	G8P Chr-1	G13P Chr-1	G18P Chr-1	G13,18P Chr-1	Norfloxacin	Gentamicin
*Escherichia coli (ATCC 9637)*	3.4	3.3	3	3	6.8	0.15	5
*Escherichia coli (ATCC 25922)*	3	4.1	5	3	4	0.3	2.5
*Pseudomonas aeruginosa (ATCC BAA-427)*	6.8	6.8	6.8	6.8	13.6	1.22	1.35
*Klebsiella pneumoniae (ATCC 27736)*	3	3.3	6.8	13.6	27.2	0.15	1.35
**Gram-Positive Bacteria**							
*Bacillus subtilis (ATCC 6633)*	6	3	3	3	3	1.2	1.2
*Staphylococcus aureus (ATCC 25923)*	3	3	3	3	3	1.22	0.67
*Staphylococcus aureus (ATCC 700698 MRSA)*	3	1.6	3	3	3	>**156**	>**86.8**
*Staphylococcus aureus (ATCC 700699 MRSA)*	1.6	1.6	1.6	3	3	>**156**	>**86.8**
*Staphylococcus aureus (BAA-44)MDR*	1.6	1.6	1.6	3	3	**39.14**	>**86.8**
*Staphylococcus aureus (ATCC 29213)*	1.6	1.6	1.6	6.8	3	2.44	1.35
*Staphylococcus aureus (ATCC 33592)Methicillin resistantGenatmicin resistant*	1.6	0.8	1.6	1.6	1.6	1.22	>**86.8**
**Fungi**						**Fluconazole**	
*Candida albicans (ATCC 10231)*	6.8	3	13.6	13.6	>30	3.26	
*Cryptococcus neoformans*	3	6.8	13.6	13.6	>30	6.53	
*Candida parapsilosis (ATCC 22019)*	3	6.8	6.8	13.6	>30	6.53	
*Sporothrix schenckii (Patient isolates)*	13.6	13.6	27.2	27.2	>30	6.53	

### The proline substituted analogs of Chrysophsin-1 induced significant damages to the membrane of *E. coli* but not to hRBCs

**Figure 2:**
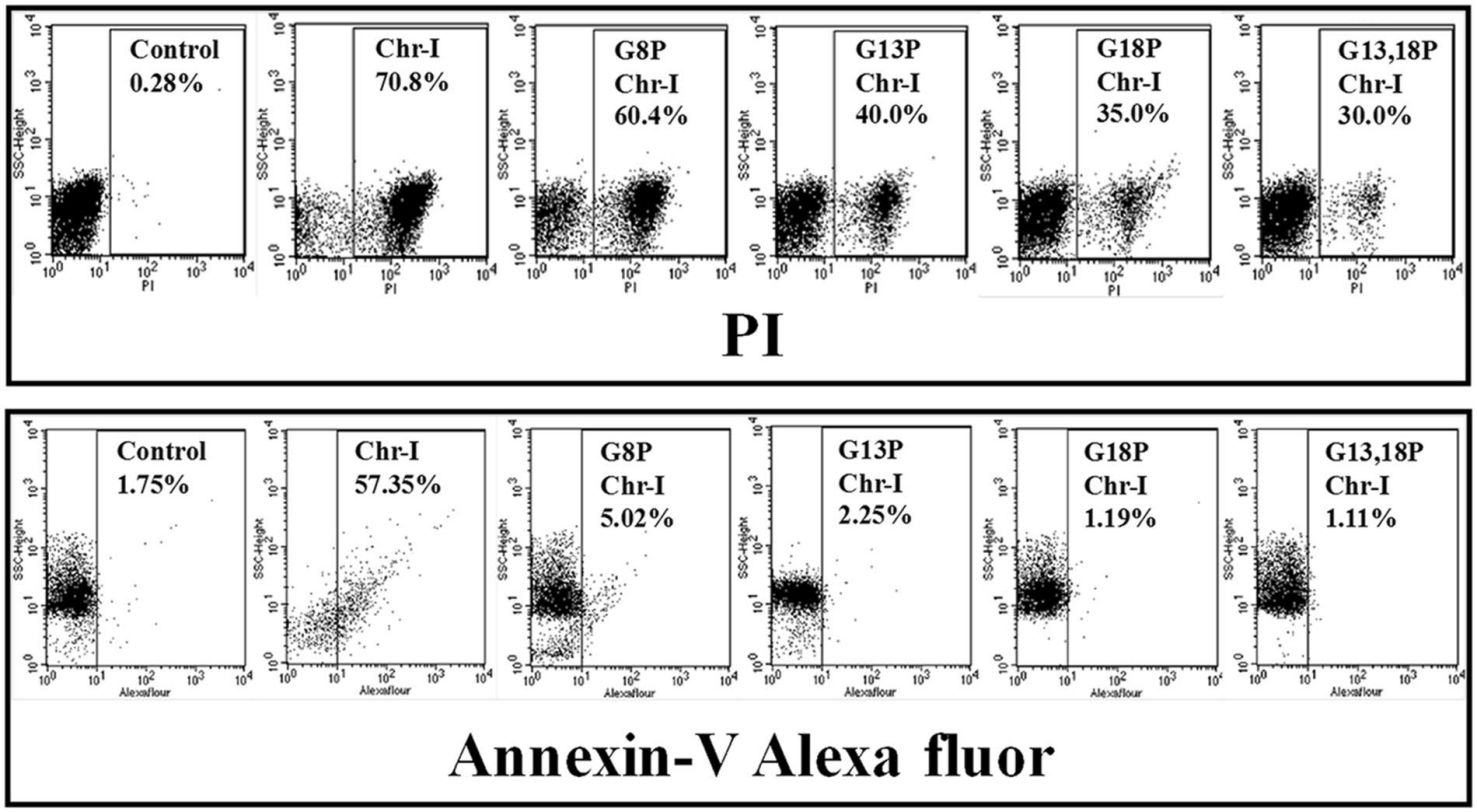
Determination of peptide-induced membrane damage of *E. coli* (ATCC 25922) cells and hRBCs by Chrysophsin-1 and its analogs. In the upper panel, the ctrl dot blot shows the autofluorescent *E. coli* cells. Left quadrant depicts the unstained bacterial cells whereas right quadrant depicts PI-stained cells. Concentrations of the peptides were 5 μM. In the lower panel, left quadrant of each blot represents unstained hRBC cells, whereas the lower right quadrant depicts the stained hRBC cells. Concentration of each of the peptides was 25 μM. 10,000 events were recorded for each sample.

Propidium iodide (PI) is often used as a marker to discriminate between the intact and ruptured cell membrane. In the upper panel, Fig. 2, the bacterial cells, not treated with any peptide did not show any staining of propidium iodide while the peptide treated cells showed significant population of PI^+^ cells. However, the selective probing of phosphatidylserine (PS) of hRBCs with membrane impermeable dye annexinV-Alexa fluor indicated that chrysophsin-1 extensively damaged the phospholipid asymmetry of hRBC membrane whereas the hRBCs treated with its variants retained it (Fig. 2 lower panel). The data were also in accord to the hemolytic activity of chrysophsin-1 and its variants.

### Salt, serum and thermal resistance of Chrysophsin-1 and its variants

The efficacy of antimicrobial peptides to clear the bacteria is highly antagonized by the physiological salts present in the body and serum of host and hence derivatization from natural peptides have been endeavoured^12^. We examined the antimicrobial activity of Chrysophsin-1 and its variants in presence of various monovalent (NaCl, KCl and NH_4_Cl) and divalent/trivalent (MgCl_2_, ZnCl_2_ and FeCl_3_) cations at their human physiological concentrations. Despite an increase in MIC values, Chrysophsin-1 and its analogs appreciably retained their antimicrobial activity in the presence of these salts (Table 4). Besides these, we also determined the antibacterial activity of Chrysophsin-1 and its variants in the presence of 10% fetal bovine serum. It has been widely observed that AMPs possess low stability when injected in the living system and are prone to degradation by both endogenous human proteases and pathogen protease secretions^13, 14^. Chrysophsin-1 and its variants also retained their antibacterial properties at high temperatures (Table 5) and displayed synergy in all peptide-streptomycin combinations against *E. coli* ATCC 25922 whereas with chloramphenicol, Chr-1, G8P-Chr-1 and G13P-Chr-1 showed synergy. On the other hand, G18P-Chr-1 and the double proline-substituted analog, G13,18P-Chr-1 showed additivity with chloramphenicol against *E. coli*. However, chrysophsin-1 and all its variants showed additive effects in combination with both Streptomycin and chloramphenicol against *S. aureus* ATCC 25923 (Table 6).

**Table 4:** Antibacterial activity of Chr-1 and its variants (in μM) in the presence of different salts at their physiological concentrations against *E. coli* ATCC 25922.

Peptide	NaCl	KCl	MgCl_2_	NH_4_Cl	ZnCl_2_	FeCl_3_	10% FBS
Chr-1	8.3	4.3	8.6	4.3	4.3	4.3	17.2
G8P-Chr-1	8.6	4.3	4.3	2.0	2	4.1	8.6
G13P-Chr-1	8.6	4.3	4.3	2.0	4	4.1	8.6
G18P-Chr-1	4.3	4.3	8.6	4.3	6.6	4.1	17.2
G13,18P-Chr-1	20	6	20	5	12	26	>33

The final concentrations of NaCl, KCl, NH_4_Cl, MgCl_2_, ZnCl_2_, and FeCl_3_ were 150 mM, 4.5 mM, 6 μM, 1mM, 8 μM and 4 μM respectively.

**Table 5:** Antibacterial activity of Chr-1 and its variants (in μM) against *E. coli* and *S. aureus* after incubating at different temperatures.

Peptide	*E. coli* (ATCC 25922)			*S. aureus* (ATCC 25923)		
	4 °C	60 °C	90 °C	4 °C	60 °C	90 °C
Chr-1	3	8.3	8.6	3	12.6	16.6
G8P-Chr-1	4.1	4.3	8.3	3	4.3	8.6
G13P-Chr-1	5	8.6	12.3	3	13	17.3
G18P-Chr-1	3	8.3	17.2	3	33	33
G13,18P-Chr-1	4	14	17.2	3	>33	>33

**Table 6:** Synergy experiments of Chr-1 and its variants in combination with Streptomycin and Chloramphenicol against *E. coli and S. aureus* as expressed in terms of their FICI values.

Peptide	*E. coli* ATCC 25922		*S. aureus* ATCC 25923	
	Streptomycin	Chloramphenicol	Streptomycin	Chloramphenicol
Chr-1	0.43	0.48	0.83	0.51
G8P-Chr-1	0.43	0.35	0.83	0.76
G13P-Chr-1	0.43	0.48	0.83	0.76
G18P-Chr-1	0.49	0.75	0.83	0.83
G13,18P-Chr-1	0.49	0.75	0.83	0.83

The MIC values of Chloramphenicol and Streptomycin were 8 μM and 2 μM respectively against *E. coli* ATCC 25922, and their MICs against *S. aureus* ATCC 25923 were 32 μM and 8 μM.

### Chrysophsin-1 and its analogs induced permeabilization of bacterial and mammalian membrane mimetic lipid vesicles at a varying degree as evident from biophysical assays

**Figure 3:**
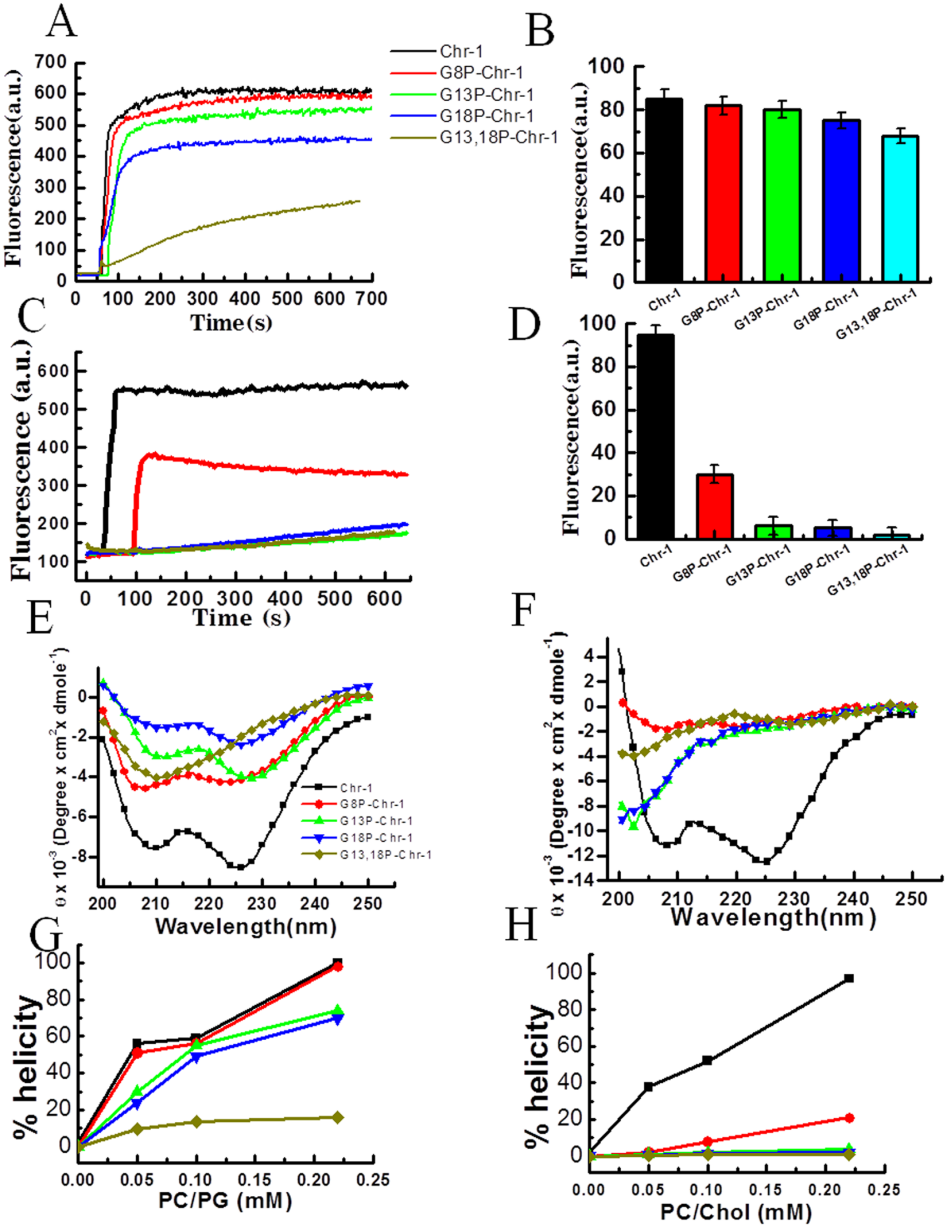
Determination of the diffusion potential and estimation of secondary structures of Chrysophsin-1 and its variants in lipid vesicles of different compositions. (**A**) Representative fluorescence profiles for different peptide-induced depolarization of PC/PG [3:1 (w/w)] vesicles at 8 μM concentration for each of the peptides. (**B**) Shows the percent fluorescence recovery in [PC/PG 3:1 (w/w)]. (**C**) Represents fluorescence profiles for peptide-induced depolarization of PC/Chol [(8:1(w/w)] vesicles at 15 μM concentration for each of these peptides. (**E** and **F**) Show the CD spectra of Chrysophsin-1 and its variants in PC/PG and PC/Chol lipid vesicles respectively at 25 μM concentration of each peptide. (**G** and **H**) Depict the increment in the helicity of the peptides with increasing concentrations of PC/PG and PC/Chol lipid vesicles respectively. Percent helicity was calculated by the Secondary Structure Estimation (SSE) software of JASCO J-1500 Spectrometer. Symbols as indicated in (**A**).

To understand the mode of action of Chrysophsin-1 and its analogs, permeabilization of bacterial membrane mimetic, phosphatidylcholine/phosphatidylglycerol (PC/PG, 3:1 w/w) & phosphatidylethanolamine/phosphatidylglycerol (PE/PG, 7:3 w/w) and mammalian membrane mimetic, phosphatidylcholine/cholesterol (PC/Chol 8:1 w/w) lipid vesicles were determined in presence of the peptides. For this purpose, the efficacy of different peptides to dissipate diffusion potential across the lipid vesicles of both kinds was measured at a fixed peptide concentration (8 μM for PC/PG & PE/PG vesicles and 15 μM for PC/Chol vesicles) by employing a potential sensitive dye 3,3′-dipropylthiadicarbocyanine iodide. (diS-C3–5). Peptide-induced permeabilization of lipid vesicles was indicated by an increase in fluorescence of the dye (Figures 3A,C and S3A, Supplementary Information) which resulted from the dissipation of diffusion potential across the phospholipid membrane. The extent of membrane permeabilization of PC/PG and PC/Chol lipid vesicles in presence of different peptides was measured with respect to peptide-induced fluorescence recovery (Fig. 3B and D) whereas permeabilization of PE/PG vesicles in presence of these peptides has been presented in Figure S3B, Supplementary Information. Chrysophsin-1 and its analogs maintained their relative efficacy to permeabilize negatively charged PC/PG (Fig. 3A and B) and PE/PG (Figure S3A and S3B, Supplementary Information) lipid vesicles that were quite comparable. However, in mammalian membrane mimetic lipid vesicles, the native Chrysophsin-1 showed the maximum permeabilization and all its proline-substituted analogs exhibited much lower efficacy (Fig. 3C and D). The results suggested that substitution of glycine residue(s) with proline residue(s) in GXXXXG motifs of Chrysophsin-1 impaired its effectiveness to permeabilize mammalian membrane mimetic lipid vesicles while most of these analogs appreciably retained the efficacy of the native peptide to permeabilize the bacterial membrane mimetic lipid vesicles. Chrysophsins are known to adopt α-helical structures in presence of both mammalian and bacterial membrane mimetic environments^8^. In an aqueous environment, the parent peptide and its analogs remained unstructured. Circular dichroism (CD) spectra obtained for chrysophsin-1 and its variants showed characteristics of α-helical structure in PC/PG (3:1 w/w) vesicles. Helix content of the peptides enhanced with an increase in the concentration of PC/PG vesicles (Fig. 3G). The results indicated that despite the introduction of proline residue(s) which is also known as a helix breaker, Chrysophsin-1 analogs particularly its single-proline substituted analogs significantly retained the helical structure of the parent peptide in negatively charged lipid vesicles. However, a contrasting picture was seen when the CD spectra were recorded in presence of mammalian membrane mimetic, PC/Chol (8:1 w/w) lipid vesicles. With the progressive replacements of glycine residue(s) with proline residue(s), the secondary structure of the peptides in PC/Chol lipid vesicles also reduced progressively. The molar ellipticity values were strongly affected (Fig. 3F) and the helical contents of these peptides, determined by Secondary Structure Estimation (S.S.E.) software, dropped significantly (Fig. 3H) with a concomitant rise in random coil and turn conformations (data not shown).

### Visualization of interaction of Chrysophsin-1 and its analogs with *E. coli* and *S. aureus* under scanning electron microscope (SEM)

**Figure 4:**
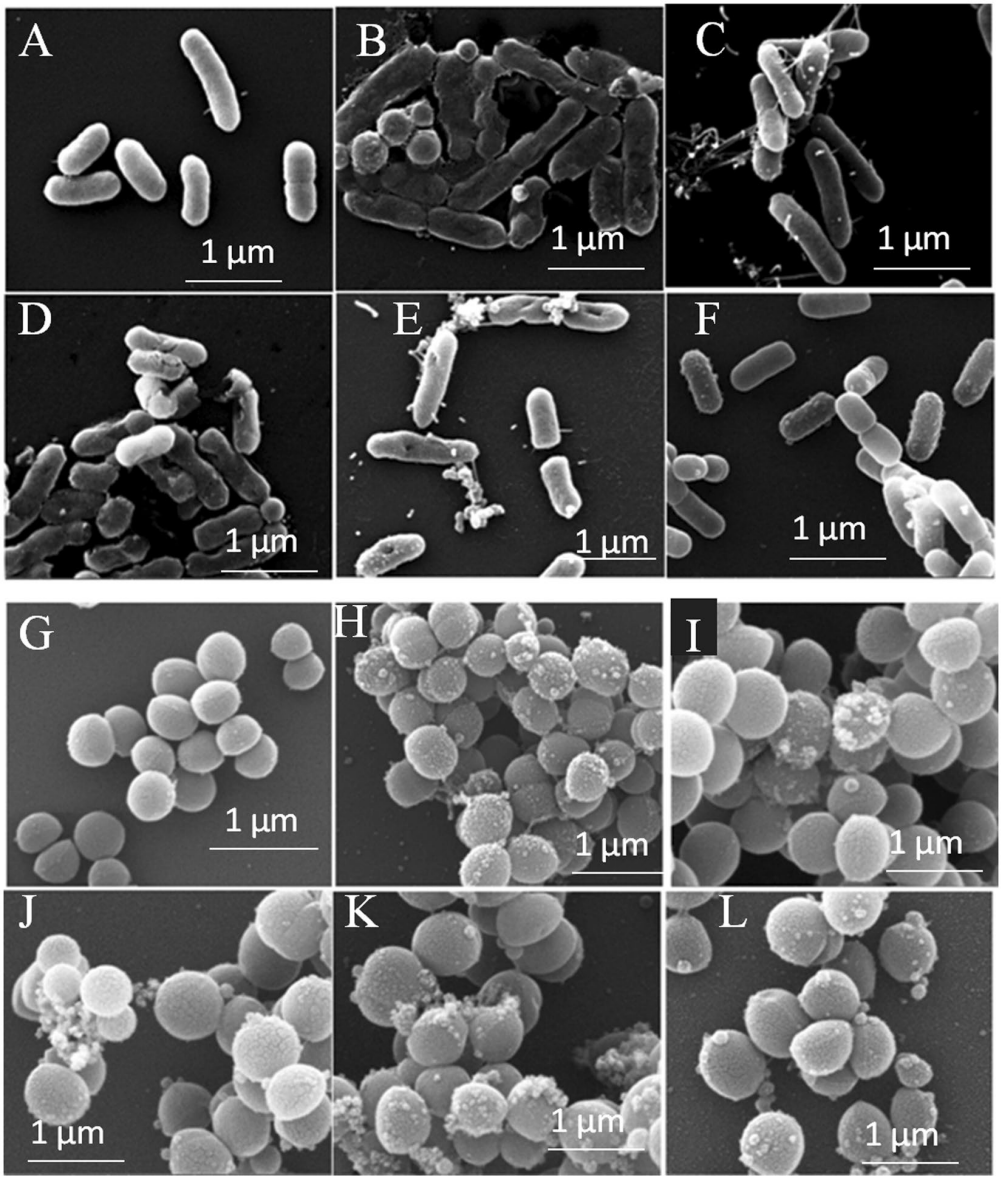
Electron microscopic visualization of the effect of Chrysophsin-1 and its analogs on *E. coli* ATCC 25922 and *S. aureus* 25923 at peptide concentrations of 2 X MIC. SEM micrographs of *E. coli*: (**A**) Control, no peptides; (**B**) Melittin-treated, positive Control; (**C**) Chr-1-treated. (**D**) G8P-Chr-1-treated (**E**) G13P-Chr-1 treated (**F**) G18P-Chr-1 treated SEM micrographs of *S. aureus*: (**G**) Control, no peptides; (**H**) Melittin-treated, positive Control; (**I**) Chr-1-treated. (**J**) G8P-Chr-1-treated (**K**) G13P-Chr-1 treated (**L**) G18P-Chr-1 treated.

To obtain further evidence for the mechanism of antimicrobial activity of these peptides, morphology of Gram-negative, *E. coli* ATCC 25922 and Gram-positive, *S. aureus* ATCC 25923, was visualized under SEM following their treatments with Chrysophsin-1 and its selected analogs. Both the bacteria without any treatment of peptide appeared healthy with a smooth surface and proper shape. However, images of the peptide-treated cells showed distinct damage to the membrane organization of both *E. coli* and *S. aureus* that include blisters, protruding bubbles and burst cells (Fig. 4). Bacteria were treated with melittin, a well-known membrane-damaging AMP and the SEM images were used as positive controls.

### Tryptophan Fluorescence studies of Chrysophsin-1 and its variants in lipid vesicles that mimic bacterial and mammalian cell membranes

**Figure 5:**
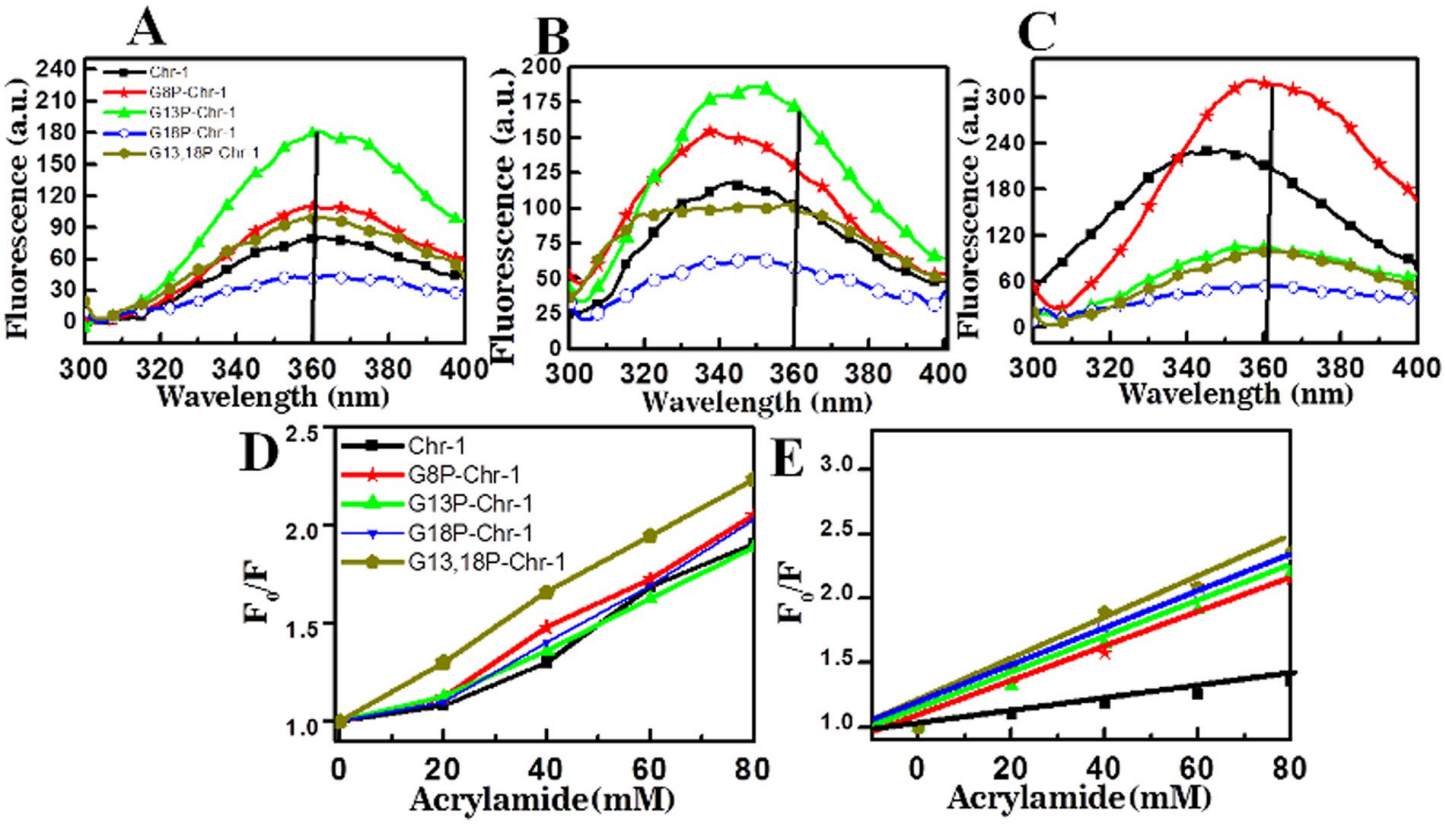
Intrinsic tryptophan fluorescence of Trp4 of Chr-1 and its analogs in different environments. (**A**) Shows the inherent fluorescence of all the peptides in Phosphate buffered saline (PBS) (pH-7.4). (**B**) Shows the blue shift of the peptides (3 µM) when they were added to the PBS with subsequent addition of negatively charged PC/PG vesicles (165 µM). (**C**) Shows the blue shift of the peptides (10 µM) when they were added to the PBS with subsequent addition of zwitterionic PC/Chol vesicles (220 µM). (**D** and **E**) Shows the Stern-Volmer plots to illustrate the effect of adding an aqueous acrylamide quencher to reveals the peptides interaction with PC/PG and PC/Chol vesicles respectively. Symbols as indicated inside the different panels.

The intrinsic fluorescence of tryptophan residue located at the 4^th^ position of Chrysophsin-1 can be employed as a reporter of the environment sensed by it^8^ and its variants when they interact with lipid vesicles of different compositions. Fluorescence spectra of both chrysophsin-1 and its variants in aqueous environments were characterized by fluorescence maxima at 360 nm (Fig. 5A). However, following the addition of PC/PG vesicles, tryptophan emission maxima of these peptides shifted to shorter wavelengths (~20 nm) with increased intensity (Fig. 5B). A contrasting picture was observed when the same experiment was performed with PC/Chol vesicles (Fig. 5C). Except Chr-1, the proline-substituted variants did not show any significant tryptophan shift in PC/Chol lipid vesicles indicating weaker interaction of Chrysophsin-1 with mammalian membrane mimetic model membrane following the substitution of a proline residue in its GXXXXG motif (Fig. 5C). To obtain further evidence for the localization of tryptophan residues of Chrysophsin-1 and its analogs in PC/PG and PC/Chol lipid vesicles acrylamide quenching of their tryptophan fluorescence was studied in presence of these lipid vesicles. In PC/PG vesicles, Chrysophsin-1 and its analogs except G13,18P-Chr-1 exhibited comparable quenching of tryptophan fluorescence. The results suggested that tryptophan residues of Chrysophsin-1 and its single proline substituted analogs were very similarly located within the hydrophobic milieu of PC/PG vesicles and hence not accessible by aqueous quencher acrylamide (Fig. 5D). However, the tryptophan residue of the double proline substituted analog, G13,18P-Chr-1 showed higher accessibility to acrylamide indicating its weaker interaction with PC/PG vesicles which could be further implicated in its lower antibacterial activities. Interestingly in zwitterionic PC/Chol lipid vesicles, the native Chrysophsin-1 showed the lowest accessibility indicating its strongest interaction with this kind of lipid vesicles (Fig. 5E). However, the proline-substituted analogs showed appreciably higher interaction with acrylamide suggesting that their tryptophan residues were exposed to the aqueous environment due to their weaker interaction with PC/Chol vesicles (Fig. 5E).

### **Inhibition of production of pro-inflammatory cytokines in human monocyte, THP-1 cells**

**Figure 6:**
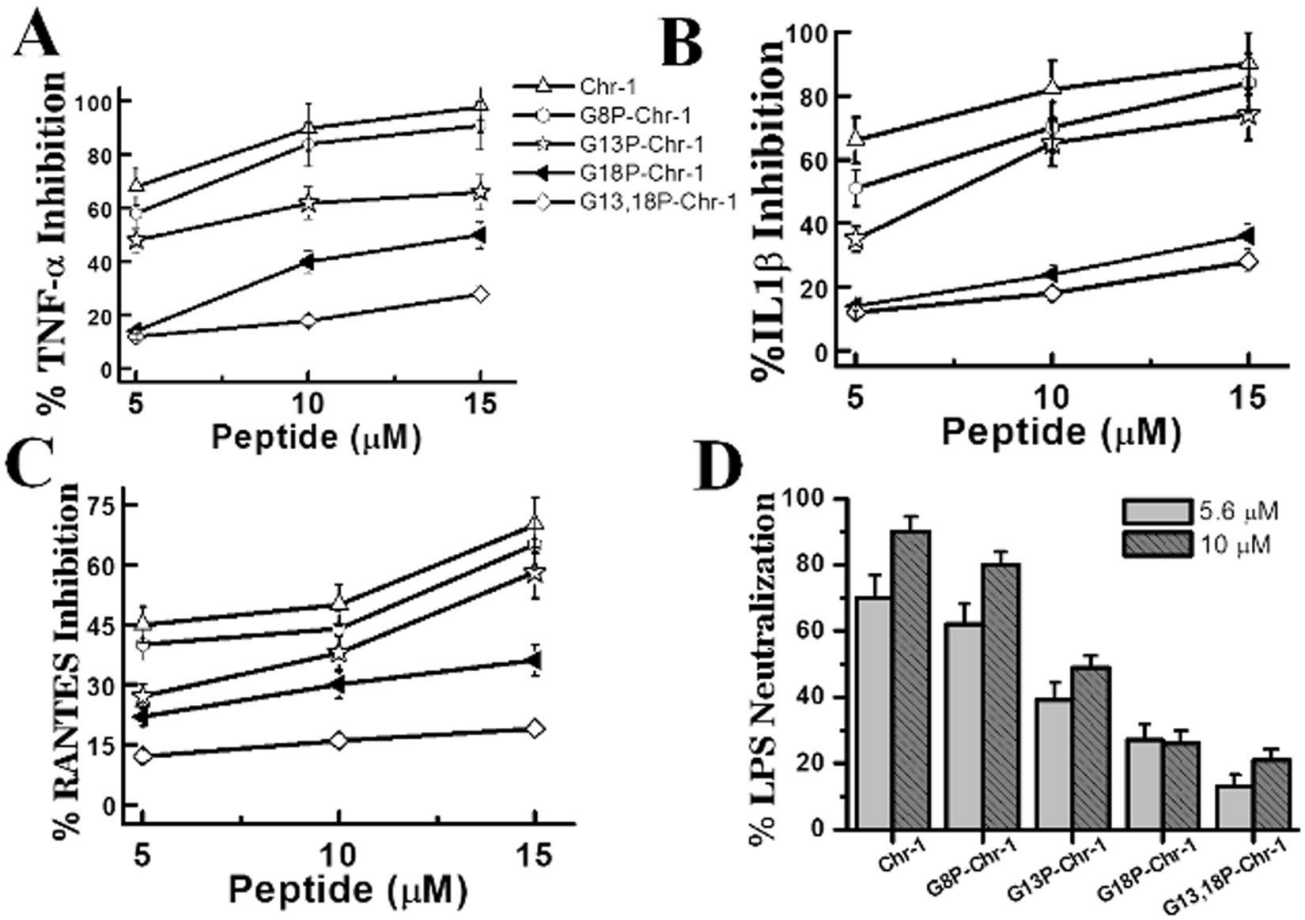
Effect of Chrysophsin-1 and its variants on the production of TNF-α in LPS-stimulated human monocytes: (**A**) Percentage inhibition of TNF-α production in LPS-stimulated THP-1 cells in concentration dependent manner. (**B**) IL1β inhibition by the peptides. (**C**) Dose-dependent inhibition of RANTES in response to LPS with increasing peptide concentrations. (**D**) LAL assay of peptides to show their binding to LPS at two different concentrations. Symbols as indicated in A.

Chrysophsin-1 can inhibit LPS-mediated TNF-α production in RAW 264.7 murine macrophage cell line^15^. However, the basis of this inhibition and the effect of Chrysophsin-1 on the LPS-induced production of other pro-inflammatory cytokines were not known. Further, considering cytotoxic nature of Chrysophsin-1, its anti-endotoxin property possesses very little future application potential. We observed that both Chrysophsin-1 and its analogs were able to inhibit the LPS-mediated production of pro-inflammatory cytokines/chemokines namely, TNF-α, IL-1β and RANTES in a dose-dependent manner at concentrations between 5–15 μM (Fig. 6A–D). Limulus amebocyte lysate (LAL) assay was performed to show that all the peptides directly bound to LPS and inhibited the downstream signaling responsible for the production of pro-inflammatory cytokines.

### Western Blot Analysis to show the inhibition of the nuclear translocation of NF-κB

**Figure 7:**
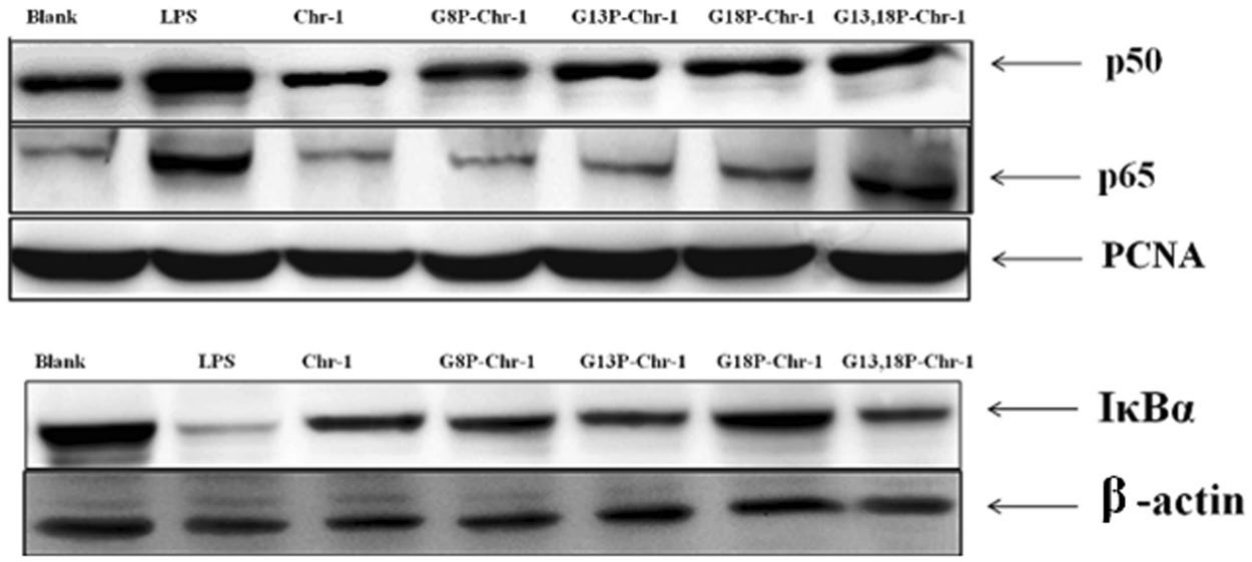
Determination of expression level of p50, p65 and IκBα in LPS-stimulated PMA-treated THP-1 cells in the presence of Chrysophsin-1 and its analogs. PCNA was used as internal nuclear loading control. β-actin used as internal control for IκBα. ctrl stand for untreated cells. Peptide concentration was 10 μM and LPS 100 ngml^−1^.

Inflammation stems from the translocation of two subunits of NF-κB namely p50 and p65^16^. In PMA primed THP-1 cells, we observed an inhibition of the LPS mediated nuclear translocation of NF-κB in the presence of Chrysophsin-1 and its analogs indicating that the genes responsible for the transcription of TNF, IL1β, RANTES etc are not activated which further may be the reason for reduced expression of pro-inflammatory mediators in ELISA experiments. Additional, evidence of inhibition of NF-κB came from the immunoblotting experiments of IκBα (Fig. 7). The latter holds the p50 and p65 subunits in the cytoplasm of the resting cells. The immunoblots showed that IκBα did not undergo proteasomal degradation when LPS-stimulated THP-1 cells were treated with Chrysophsin-1-analogs and thus NF-κB subunits remained in the cytoplasm of these cells.

### *In vivo* anti-endotoxin properties of non-toxic analog of Chrysophsin-1 namely G13P-Chr-1

**Figure 8:**
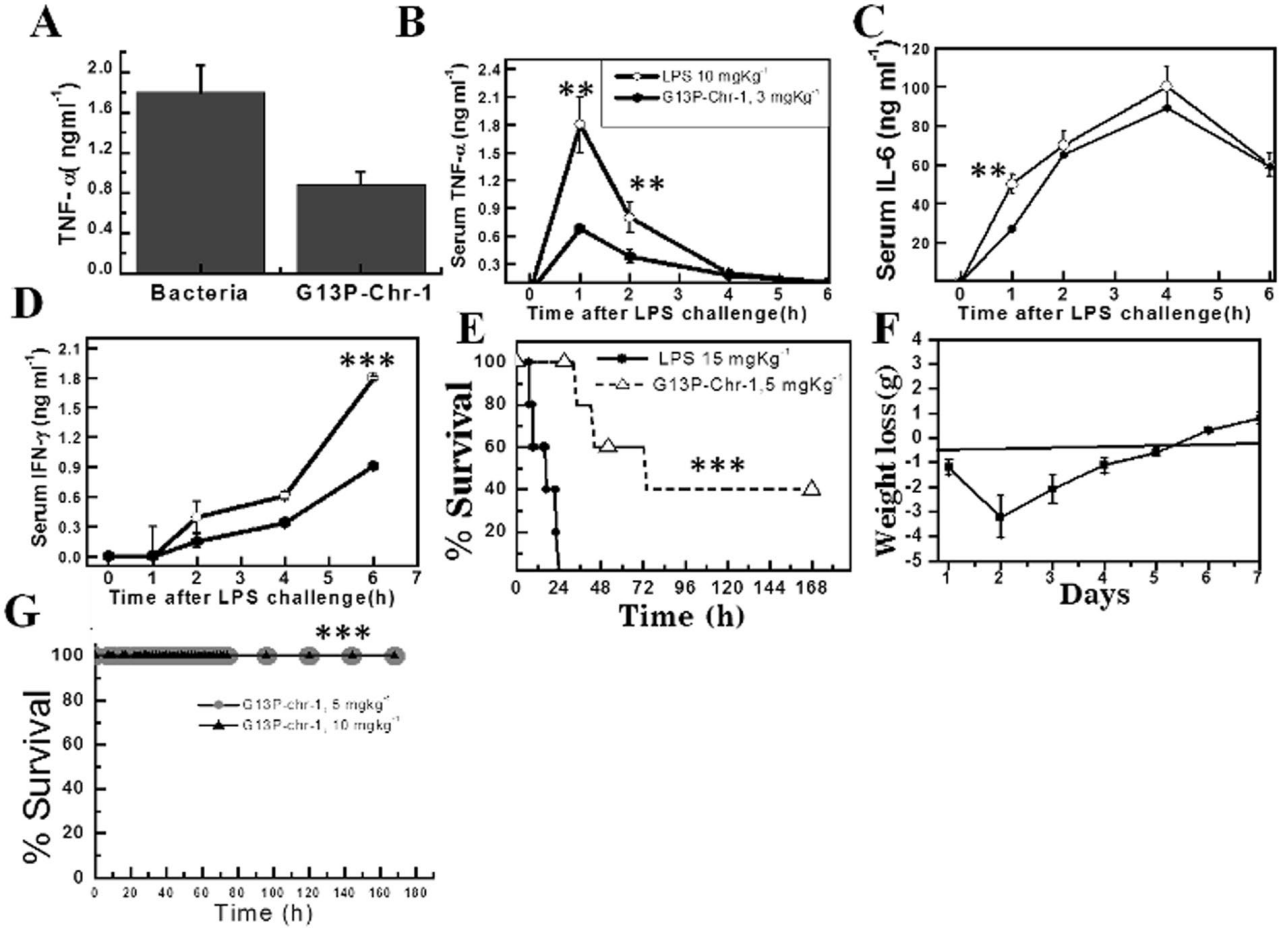
*In vivo* anti-endotoxin activities of G13P-Chr-1. (**A**) Inhibition of TNF-α production two hours post infection to mice with mid-logarithmic phase *E. coli* ATCC 25922. (**B**) Serum levels of at different time points when mice were injected with indicated concentrations of LPS and peptides. (**C**) and Panel **D**, Serum levels of IL-6 and IFN-γ at various time points. (**E**) Anti-LPS survival assay of G13P-Chr-1 to show its protective effects. LPS was injected intraperitoneally followed by peptides treatment. (**F**) Plot of weight loss (g) of G13P-Chr-1 peptide-treated mice, challenged with LPS with respect to the number of experimental days. No animals survived in the peptide-untreated group that were challenged with LPS (after day 2); hence the weight curve for peptide-untreated mice is not shown. (**G**) *In vivo* toxicity of G13P-Chr-1. BALB/c mice were injected intraperitoneally with the peptide (5 and 10 mgkg^−1^ of body weight). All these mice were alive after 7 days. Symbols, as indicated in B. The quantity of cytokines, were obtained by the standard curve for each cytokine (**A**–**D**). Error bars represent the possible amount of error in each dataset (**A**–**D**). P value was determined by the log-rank test. (***P < 0.001, **P < 0.01).

G13P-Chr-1 possessed equal potency in killing bacteria as the parent peptide and was significantly non-toxic in nature. Besides this, it also retained its antibacterial activities in physiological salt concentrations, serum and at higher temperatures (i.e. at 60 °C and 90 °C), well beyond the physiological body temperature of mammals (37 °C). Taking into considerations of all these *in vitro* results, we further examined the *in vivo* activities of this peptide in mice. In the first experiment, we directly injected the mid-logarithmic phase bacteria *E. coli* ATCC 25922 in BALB/c mice and these mice were either untreated or treated with G13P-Chr-1. Serum was isolated from the blood obtained by bleeding the mice retro-orbitally two hours post infection and ELISA was performed to determine the amount of TNF-α produced in the untreated group and the peptide-treated group. There was a significant reduction in TNF- α production when *E. coli* ATCC 25922 injected mice were further treated with one of the non-toxic Chrysophsin-1-variants G13P-Chr-1 (Fig. 8A). The experiment was further performed with commercially available LPS in which one group of mice were challenged with LPS (10 mgKg^−1^) while the other group was treated with G13P-Chr-1 peptide (3 mgKg^−1^) and LPS (10 mgKg^−1^). Blood was isolated at four different time points 1 h, 2 h, 4 h and 6 h and was subjected to ELISA after dilutions. A significant reduction in LPS-induced production of pro-inflammatory cytokines like TNF-α, IL-6 and IFN-γ in the serum of mice group, treated with both peptide and LPS was observed in comparison to the mice group which was challenged with LPS only.

### *In vivo* survival of mice against LPS-challenge in presence of G13P-Chr-1

The mice group treated with both G13P-Chr-1 and LPS showed 40% survival in a 7 days experiment following LPS and peptide injection. Initially, the peptide-treated group of mice showed signs of sepsis similar to the only LPS-treated group but gradually some of them recovered from the symptoms because of the anti-LPS properties of G13P-Chr-1. The weight of the recovered mice was minimum post 48 h of LPS challenge. The gain in weight preceded the recovery of survived mice. *In vivo* cytotoxicity of G13P-Chr-1 was examined by administering 5 and 10 mgKg^−1^ of this peptide to a group of BALB/c mice comprising five animals and their survival was monitored for seven days. 100% survival of mice was observed suggesting no *in vivo* cytotoxicity of this peptide (Fig. 8G).

## Discussion

The key finding of this study is the identification of GXXXXG motifs in Chrysophsin-1 for the first time and introduction of small amino acid substitution in this motif to yield non-toxic variants of this highly cytotoxic AMP without compromising its antimicrobial and anti-endotoxin activities. This motif is also present in several other antimicrobial peptides as listed in Table S2, Supplementary Information. The motifs formed by double glycine residues like GXXG and GXXXG have been characterized that seem to play a critical role in transmembrane helix interactions^17, 18^. GXXXG motifs are found in transmembrane proteins as helices^19^ and also in soluble proteins where they aid in helix-helix interactions^20^. The replacement of glycine residue(s) in GXXXXG motif with alanine and valine residue(s) did not evoke significant effect on the cytotoxicity of chrysophsin-1 (Figure S2, Supplementary Information) or on its secondary structure in mammalian membrane mimetic, zwitterionic PC/Chol lipid vesicles (Figure S4, Supplementary Information). The results indicated towards the importance of a helix breaker like proline in impairing the structural property of this motif in mammalian membrane mimetic environments. Since proline is the smallest amino acid lacking a side chain, any amino acid other than proline in place of glycine would have altered the hydrophobicity of the resulting analog which is an important attribute in antimicrobial peptides^21^. Moreover, glycine residue(s) have been replaced with proline residues earlier without causing much secondary structural changes^22, 23^. Thus by designing the analogs in this way, the net positive charge and percent hydrophobicity was kept equivalent to the native peptide, Chrysophsin-1 (Table 2). The toxicity studies showed that there was a remarkable reduction in the Chrysophsin-1 induced hemolysis of hRBCs after introducing proline residue(s) in it (Fig. 1). The FACS studies also supported the cell selectivity of Chrysophsin-1 analogs towards bacterial membrane over erythrocytes membrane. All the peptides ruptured the bacterial cell membrane as there was a significant bacterial population which was stained with PI indicating their compromised cell membranes. Contrary to this, in annexin V staining of human red blood cells, only Chrysophsin-1 treated RBCs lost their normal phospholipid asymmetry whereas the other proline substituted analogs retained it (Fig. 2). Though proline is known as a helix breaker, it has been shown that helix propensity of proline-containing peptides mostly depends on the environment of such peptides^24^. Indeed, proline-containing chrysophsin-1 analogs failed to adopt a significant helical structure in presence of zwitterionic lipid vesicles but adopted an appreciable helical structure in negatively charged lipid vesicles (Fig. 3A and B).

However, Chrysophsin-1 as well as its non-cytotoxic variants showed strong antibacterial activity against both Gram-negative and Gram-positive bacteria. Besides, the proline substituted analogs were also active against MRSA and MDR strains of *S. aureus* (Table 3). Results indicated that the alteration in GXXXXG motif did not impede the anti-bacterial activities of Chrysophsin-1 presumably because of other biophysical properties like positive charge; hydrophobicity and amphipathicity were mostly unaltered after the amino acid substitutions in this motif (Figure S5, Supplementary Information). Chrysophsin-1 and its analogs appreciably retained their antibacterial activity in elevated salt concentrations, in the presence of serum and at increased temperature (Table 5). One possible explanation of their antibacterial activity in presence of salt could be their marine origin. Chrysophsins evolved in an environment containing extremely high salt concentrations and diverse ecological niches^7^.

Chrysophsin-1 and all its variants showed synergy with streptomycin against *E. coli* ATCC 25922 whereas with chloramphenicol, except G18P-Chr-1 and G13,18P-Chr-1 all other peptides displayed synergy (Table 6). However, all these peptides showed additive effect with both Streptomycin and chloramphenicol against *S. aureus* ATCC 25923. Thus overall, of the ten peptide-antibiotics combinations against *E. coli*, eight showed synergistic effects while all the ten peptide-antibiotics combinations against *S. aureus* were additive in nature with no synergy. The synergy trait of antimicrobial peptides is an important characteristic as it allows better intracellular uptake of antibiotics that subsequently have improved bactericidal effects^25^. The variants of Chrysophsin-1 excluding the double proline-substituted G13,18P-Chr-1 also displayed appreciable anti-fungal activities against different fungi tested. This is of significance due to the fact that peptide-based antifungal therapies are an emerging field of research^26, 27^.

The results also indicated that the proline-substituted analogs interacted differently with the negatively charged, bacterial (PC/PG, 3:1 & PE/PG, 7:3 both w/w) and zwitterionic, mammalian (PC/Chol, 8:1 w/w) model membranes as evident from the contrasting differences in permeabilization of these lipid vesicles in presence of the peptides.

The mode of action of Chr-1 and its analogs against microorganisms was further verified by taking images of Gram-positive bacteria, *S. aureus* ATCC 25923 and Gram-negative bacteria, *E. coli* ATCC 25922 under SEM following their treatment with Chr-1 and its selected variants. A critical alteration of the outer membrane organization was seen in our study for both *E. coli* and *S. aureus* treated The SEM visualizations further confirmed that the peptides targeted the membranes of these microorganisms to exert their antibacterial activity (Fig. 4). Moreover, in tryptophan shift experiments a higher shift (~20 nm) of tryptophan emission maxima of Chr-1 and its proline substituted analogs in PC/PG vesicles suggested that these peptides interacted strongly with negatively charged lipid vesicles resulting in the localization of their tryptophan residues in a more hydrophobic environment. However, in mammalian membrane mimetic, PC/Chol lipid vesicles only Chrysophsin-1 showed a significant blue shift a (~19 nm) while its variants did not show any significant shift of tryptophan emission maxima. The recorded spectra were very similar to that obtained in PBS only (Fig. 5). It is most likely that Chrysophsin-1 variants interacted weakly with PC/Chol lipid vesicles leaving their tryptophan residues exposed onto the polar aqueous environment. The Stern-Volmer plots indicated a reduced accessibility of Chrysophsin-1 to the aqueous quencher acrylamide in both negatively charged, PC/PG and zwitterionic PC/Chol lipid vesicles which indicated its strong interactions with both of these kinds of lipid vesicles and may further verify the reason of its strong antimicrobial and cytotoxic properties. However, the Chrysophsin-1 variants with substitutions in GXXXXG motif showed far more accessibility to acrylamide in PC/Chol environment which is indicative of weaker penetration of their Trp residues when they interacted with this kind of lipid vesicles. In contrast, in PC/PG lipid vesicles the Stern-Volmer plots revealed lesser accessibility of tryptophan residues of Chrysophsin-1 analogs to acrylamide that was comparable to the native peptide, Chrysophsin-1 suggesting that despite proline substitutions in GXXXXG motif the analogs maintained the interaction of their parent peptide with negatively charged lipid vesicles. Thus, the results indicated that glycine residues of GXXXXG motifs play a more crucial role in interaction with hydrophobic PC/Chol lipid vesicles than in negatively charged PC/PG vesicles. Though the present study demonstrated that glycine to proline substitution in GXXXXG motif drastically reduced the cytotoxicity of chrysophsin-1, the underlying molecular basis remained unknown. Induction of helical structure and oligomerization of AMPs are believed to be crucial steps for forming pores in bilayer lipid membrane and showing their biological activities^28^. Chrysophsin-1 adopted a significant helical structure in presence of mammalian membrane mimetic, zwitterionic lipid vesicles however the substitution of glycine with proline residue(s) in its GXXXXG motif significantly abrogated its helical structure in the same environment. We speculate that the identified GXXXXG motifs could assist chrysophsin-1 to oligomerize and form pores in the mammalian membrane and therefore may play a crucial role in its cytotoxic properties^29^.

The elevated levels of LPS induced pro-inflammatory cytokines production in clinical conditions like sepsis may lead to consumption of huge health care resources and even mortality in septic shock cases^30^. Chrysophsin-1 and its analogs induced a marked reduction in LPS-mediated production of important pro-inflammatory cytokines like TNF-α, IL-1β and IFN-γ in human monocyte cell line THP-1 in a concentration-dependent manner (Fig. 6). It was also observed Nitric oxide (NO), a mediator of inflammation and TNF-α was also inhibited in mice murine macrophages (Figure S6, Supplementary Information). The alteration of GXXXXG motif in Chrysophsin-1 did not compromise with its anti-endotoxin properties since the single proline-substituted variants retained the anti-LPS properties of the parent peptide well below the concentrations at which they could cause any toxicity to hRBCs. LAL assay and biophysical assays like CD experiments with LPS (Figure S7, Supplementary Information) indicated that Chrysophsin-1 and its variants bind directly to lipopolysaccharide and can neutralize its toxicity. To confirm that anti-endotoxin property of these peptides was solely because of immunomodulation and not due to toxicity effects, we performed the western blot assays to show the inhibition of the nuclear translocation of NF-κB subunits p65 and p50 in PMA primed THP-1 cells. We observed that majority of the NF-κB subunits p65 and p50 were retained in the cytoplasm when cells were treated with chrysophsin-1 or its variants and LPS (Fig. 7). This suggests that peptides diminished the influence of LPS as the peptide treated cells retained NF-κB inside the cytoplasm which explains the reduced production of pro-inflammatory cytokines.

To verify the possibilities of using Chrysophsin-1 variants as anti-LPS infectives we performed *in vivo* experiments with the non-cytotoxic analog of Chrysophsin-1, G13P-Chr-1 which showed appreciable efficacy to neutralize the LPS-induced production of pro-inflammatory cytokines in mice. The other peptides were not included in *in vivo* studies either due to high cytotoxicity or due to low anti-endotoxin activity. The protective nature of G13P-Chr-1 was further confirmed when we performed the survival assay of mice challenged with LPS in a model of severe sepsis. At a lethal dose of 15 mgKg^−1^, all the control mice of only LPS group died within 24 h of LPS injection whereas the mice group treated with 5 mgKg^−1^ of G13P-Chr-1 along with the same dose of LPS showed 40% survival. Further, G13P-Chr-1 did not exhibit any cytotoxicity in the *in vivo* experiments with mice (Fig. 8G). Given the fact that mortality due to sepsis in very high in hospitals and ICU settings^31^, G13P-Chr-1 could be a lead molecule in this field.

Thus by substituting glycine residues with proline residues in the GXXXXG motif of Chrysophsin-1 we were able to develop considerably non-cytotoxic, salt, serum and heat stable analogs that displayed significant antimicrobial activities against variety of microorganisms including bacteria, fungi and resistant strains of *S. aureus* and inhibited the LPS-induced production of pro-inflammatory cytokines in human monocytic cell line, THP-1. Further, one of the Chrysophsin-1-analogs exhibited significant efficacy to prevent the death of mice against a lethal dose of LPS confirming its pharmacological potential. Thus overall the results demonstrated the identification and characterization of GXXXXG motif in Chrysophsin-1 with its implication in the design of its cell-selective analogs without compromising its antimicrobial and antiendotoxin properties. The results could be useful for the design of totally novel anti-microbial and anti-endotoxin peptides by employing this GXXXXG motif.

## Materials and Methods

### Materials

Rink amide MBHA resin (loading capacity: 0.36–0.78 mmol/g), N-α-Fmoc and necessary side-chain protected amino acids were purchased from Novabiochem. Coupling reagents for peptide synthesis including Oxyma Pure [ethyl 2-cyano-2-(hydroxyimino) acetate], HCTU [O-(6-chlorobenzotriazol-1-yl)-N,N,N′,N′-tetramethyluronium hexafluorophosphate] and PyBOP [benzotriazolyloxy-tris(pyrrolidino)-phosphonium hexafluorophosphate] were purchased from Novabiochem. DIC (N, N′-diisopropylcarbodiimide), DIPEA (N, N-diisopropylethylamine) and NMM (N-methylmorpholine) were purchased from Sigma-Aldrich, USA. Dichloromethane (DCM), N, N-dimethylformamide (DMF), piperidine, diethyl ether and trifluoroacetic acid (TFA) were purchased from Spectrochem Pvt. Ltd, India. Acetonitrile (HPLC grade) and ethanol were procured from Merck, India. Mueller Hinton broth and agar powder were purchased from Himedia, India. Egg phosphatidylcholine (PC) and egg phosphatidylglycerol (PG) were obtained from Avanti Polar Lipids, Inc., USA. while1,2-Dipalmitoyl-sn-glycero-3-phosphoethanolamine(PE),cholesterol(Chol), lipopolysaccharide *E. coli* 0111: B4, HEPES, Bovine serum albumin (B.S.A.), sodium dodecyl sulfate (SDS), valinomycin and Phorbol 12-Myristate 13-Acetate (PMA) were purchased from Sigma-Aldrich, USA. Propidium iodide (PI) and Annexin V–Alexa Fluor were purchased from Molecular Probes Eugene, Oregon, USA and Molecular probes, life technologies, USA respectively. For Cell culture, RPMI 1640, Fetal Bovine Serum, 100X Antibiotic–antimycotic and 0.25% Trypsin-EDTA (1X) were purchased from Gibco/Invitrogen. Sterile T-25 cm^2^ and T-75 cm^2^ polystyrene tissue culture flasks, 96-well plates and 6-well plates (3506) were from Corning Inc. The rests of the reagents were of analytical grade and procured from reputed local vendors; buffers were prepared in Milli-Q (USF-ELGA) water.

### Methods

#### Peptide synthesis and purification

The peptide synthesis was carried out manually employing a solid-phase method on rink amide MBHA resin and Fmoc chemistry as described previously^32^. The cleavage of peptides from resins and their purification was done by reverse-phase HPLC and the mass of the peptides was ascertained by mass spectroscopy.

#### Assay of hemolytic activity of the peptides

Fresh human red blood cells (hRBCs) were collected in the presence of an anticoagulant. The methodology of our experiment with human blood was in accordance with relevant guidelines and regulations of CSIR-Central Drug Research Institute Ethics Committee and was approved by it with approval No. CDRI/IEC/2014/A5. Further, informed consent was obtained from the healthy volunteer before collection of blood as per the guideline of our Institutional ethics committee. Freshly dissolved peptides in milli-Q at desired concentrations were added to the suspension of 4% red blood cells in PBS to the final volume of 200 µl and incubated at 37 °C for 45 minutes. For negative and positive controls hRBC in PBS (A_blank_) and in 0.2% (final concentration v/v) Triton X-100 (A_triton_) were used respectively^33, 34^. The percentage of hemolysis was calculated according to the following equation.

$${\rm{Percentage}}\,{\rm{of}}\,{\rm{hemolysis}}=[({{\rm{A}}}_{{\rm{sample}}}-{{\rm{A}}}_{{\rm{blank}}})/({{\rm{A}}}_{{\rm{triton}}}-{{\rm{A}}}_{{\rm{blank}}})]\times 100$$

#### Antibacterial Activity of the Peptides

Antibacterial assay against various Gram-positive and Gram-negative strains of bacteria was performed by the serial two-fold dilution method according to the previously reported procedure^35, 36^. The antibacterial assays were repeated thrice and the averages of these MIC values were reported.

#### Antifungal activity assay

The anti- fungal activity of peptides was performed according to the standard micro broth dilution technique as per NCCLS guidelines^37^. These plates were incubated in a moist chamber at 35 °C, and an absorbance at 492 nm was recorded on a VersaMax microplate reader after 48 h for *C. albicans* ATCC 10231 and *C. parapsilosis* ATCC 22019 and 72 h for *C. neoformans*.

#### Salt tolerance, thermal stability and serum stability of chrysophsin-1 and its variants

*E. coli* ATCC25922 was grown to 10^6^ CFU/ml in fresh MH broth with various divalent and monovalent salts at their physiological concentrations. Serial dilutions of chrysophsin-1 and its variants prepared with the same MH broth were added to the *E. coli* ATCC 25922 suspensions. The bacteria were incubated at 37 °C in a shaking incubator for 18 h and the MIC values of individual peptides against *E. coli* ATCC 25922 were determined^38^. Thermal tolerance of Chrysophsin-1 and its variants was checked by incubating the peptides at 4 °C, 60 °C and 90 °C for 1 h prior to MIC determination. The antibacterial activity in presence of serum was carried out by adding a final concentration of 10% fetal bovine serum in the assay diluents containing 0.4% BSA, 0.02% glacial acetic acid. The subsequent steps in both the experiments were consistent with the MIC determination method.

#### Synergy with conventional antibiotics

The checkerboard titration method was followed to check the synergy of peptides with other antibiotics. First, 2-fold serial dilutions of the antibiotics and antimicrobial peptides were prepared. Subsequently, 50 μl each of different concentrations of antibiotics and of antimicrobial peptides was mixed and added into 100 μl of log phase bacteria culture in a 96-well plate. The plates were then incubated at 37 °C for 24 h. The fractional inhibitory concentration (FIC) index (FICI) was calculated as follows^39^:

$$\begin{array}{rcl}{\rm{FICI}} & = & ({\rm{MIC}}\,{\rm{of}}\,{\rm{drug}}\,{\rm{A}}\,{\rm{in}}\,{\rm{combination}})/({\rm{MIC}}\,{\rm{of}}\,{\rm{drug}}\,{\rm{A}}\,{\rm{alone}})\\  &  & +({\rm{MIC}}\,{\rm{of}}\,{\rm{drug}}\,{\rm{B}}\,{\rm{in}}\,{\rm{combination}})/({\rm{MIC}}\,{\rm{of}}\,{\rm{drug}}\,{\rm{B}}):\end{array}$$

FICI < 0.5 is considered to indicate synergy; 0.5 < FICI < 1.0 is considered additive; 1.0 < FICI < 4.0 is considered indifferent; and FICI > 4.0 is considered antagonism.

#### Assay of peptide-induced dissipation of diffusion potential

The peptide-induced depolarization of the lipid bilayers representing mammalian membrane mimetic i.e. zwitterionic PC-Chol (8:1, wt/wt) and bacterial membrane mimetic i.e. negatively charged PC/PG (3:1 wt/wt) & PE/PG (7:3 wt/wt) was measured by using a potential-sensitive dye, diS-C3–5, as described in earlier reports^40, 41^.

#### Detection of peptide-induced membrane damage of hRBCs and bacterial cells

Peptide-induced damage of the hRBC membrane was determined by staining the cells (~3.0 × 10^7^ cells/ml) with Annexin V–Alexa Fluor following their treatment with Chrysophsin-1 and its analogs (25 μM) at 37 °C for 15 min. To check the peptide-induced damage to the *E. coli* plasma membrane, the cells were washed and incubated with 5 μM of all peptides for 1 h at 37 °C in a shaking incubator followed by propidium iodide (PI) (Invitrogen) staining at 4 °C for 30 min. Both sets of treatments were analyzed by a Becton Dickinson FACS Calibur flow cytometer using Cell Quest Pro software.

#### CD studies

The circular dichroism (CD) spectra of the peptides in PBS (pH 7.4) and in zwitterionic PC/Chol (8:1, wt/wt), negatively charged PC/PG (3:1 wt/wt) lipid vesicles were recorded on a Jasco J-1500 spectropolarimeter. The spectra obtained only for PC/Chol (8:1, wt/wt), PC/PG (3:1 wt/wt) was subtracted as background.

#### Tryptophan (Trp) fluorescence assays

Trp emission spectra of peptides were recorded in PBS and at varying lipid concentrations of PC/PG (3:1 w/w) and PC/Chol (8:1 w/w). The excitation wavelength was fixed at 280 nm and emission spectra were recorded between 300 and 400 nm, excitation and emission slits were kept 8 and 6 nm respectively in a Perkin-Elmer LS-55 fluorimeter. Emission spectra of the peptide-lipid complex were corrected for lipid-induced light scatterings, and blue shifts were calculated as the differences in emission maxima of Lipid-peptide complex and peptide alone. The localization of tryptophan residues of Chrysophsin-1 and its analogs in PC/PG (3:1 w/w) and PC/Chol (8:1 w/w) was determined by tryptophan quenching experiments using acrylamide as a quencher. Aliquots of acrylamide (3 M) solutions were added to the peptide-lipid complex. The values obtained were corrected for the scattering contribution of acrylamide and lipid. The data were analyzed according to the Stern-Volmer equation.

$${\rm{F}}0/{\rm{F}}=1+{\rm{Ksv}}[{\rm{Q}}]$$

where F0 and F represent the fluorescence intensities in the absence and the presence of the quencher (Q), respectively, and Ksv is the Stern-Volmer quenching constant, which is a measure of the accessibility of Trp to its quencher.

#### Scanning Electron Microscopy

Bacterial cells were fixed with 2.5% glutaraldehyde in phosphate buffer (pH 7.2). Samples were post-fixed in 1% osmium tetroxide and dehydrated through an ascending ethanol series, critical point dried and coated with Au-Pd (80520) using a Polaron E5000 sputter coater. Bacterial morphology was examined in an FEI Quanta 250 using an SE detector at an accelerating voltage of 30 kV^41, 42^. Melittin was used as a positive control to study the bacterial cell damage^35^.

#### Cell lines and animals

The human monocyte cell line THP-1 and murine cell line NIH-3T3 cell lines were supplied by the CSIR-Central Drug Research Institute (CDRI), Lucknow, India, cell line repository. The cell lines were maintained in RPMI medium supplemented with 10% fetal bovine serum and antibiotics in a humidified atmosphere containing 5% CO_2_ incubator. The animals used for the experiments were provided by the National Laboratory Animal Center, CSIR-CDRI (Lucknow, India).

#### Measurement of cytokines/chemokine expression levels in supernatant in THP-1 cells

Enzyme-linked immunosorbent assays (ELISAs) were carried out to estimate the amount of tumor necrosis factor alpha (TNF-α) and interleukin-1β (IL-1β) secreted by LPS-treated THP-1 cells in the presence of peptides after 4–6 h of incubation. The levels of these cytokines in the culture supernatant of untreated and LPS-treated cells were taken as the negative and positive respectively, to calculate the percent inhibition by the peptides. For RANTES estimation the peptide LPS incubation was kept up to 24 h. The experiments were repeated twice reported as percentage inhibition. The absorbance of LPS treated cell supernatant was taken as zero inhibition. Similar ELISAs were performed to estimate the amount of TNF-α IL-6 and IFN-γ secreted by BALB/c mouse blood, which was collected from the orbital sinus at different time points post LPS injection, by using enzyme-linked immunosorbent assay kits for mouse TNF-α (BD Biosciences), IL-6 (BD Biosciences) and IFN-γ (BD Biosciences).

#### Endotoxin neutralization (LAL) assay

The capacity of peptides to bind LPS was measured using a quantitative chromogenic Limulus amoebocyte lysate (LAL) assay (QCL-1000 kit; catalog number 50–647 U; Lonza), as reported previously^43^.

### Western experiments for nuclear translocation of NF-κB

Phorbol 12-myristate 13-acetate (PMA) treated THP1 cells (approx 2 × 10^5^) were stimulated with 100 ng/ml LPS in the presence of peptides (10 μM) in 6-well plates for 1 h^44^. LPS treated and untreated cells were taken as positive and negative control, respectively, representing the stimulated and unstimulated levels of protein expressions. Cells were harvested, washed with ice-cold PBS pH 7.4, and lysed in Laemmli buffer. Lysates were resolved by SDS-PAGE on 12% gel and then transferred to nitrocellulose membrane (Immobilon, Millipore). Signals were developed with alkaline phosphatase conjugated secondary antibodies with the help of substrate NBT/5-bromo-4-chloro-3-indolyl phosphate (calbiochem). PCNA and β-Actin served as nuclear and cytoplasmic loading controls respectively.

### *In vivo* assay for the inhibition of LPS mediated cytokine production

All the mice were divided into three experimental groups. Except the control group which was injected with PBS only, the other two groups were challenged with *E. coli* O111: B4 LPS at 10 mgkg^−1^ of body weight in the presence and in the absence of 3 mgkg^−1^ peptide. Blood was collected 1, 2, 4 and 6 h post LPS and peptide injection by the retro-orbital bleeding of mice. Serum was isolated and subjected to ELISA experiments for pro-inflammatory cytokines like TNF-α, IL-6 and IFN-γ^45^.

### Anti-LPS Survival assay

For survival assay, mice were challenged intraperitoneally with LPS at a lethal dose of 15 mgKg^−1^ *E. coli* 0111:B4^5^. Control mice were injected with an equal volume of PBS. G13P-Chr-1 at a single dose of 5 mgKg^−1^ was injected in the mice groups that were already administered with 15 mgKg^−1^ of LPS. Each group contained five mice. The mortality of mice was monitored up to 7 days and weight change was monitored every day. The peptide G13P-Chr-1 only was also injected in two groups of mice at 5 mgKg^−1^ and 10 mgKg^−1^ to check any mortality in mice by peptide only treatment.

### Statistical analysis

For statistical evaluation, the data were analyzed using Prism software (version 5; GraphPad). For survival analysis, the log-rank (Mantel-Cox) test was used to determine statistically significant differences. For all statistical analyses of P < 0.05, values were considered significant and the degrees of statistical significance are presented as ***P < 0.001, **P < 0.01 or *P < 0.05.

### Ethics Statement

All animal experiments were performed as per the institutional guidelines approved by the CSIR-Central Drug Research Institute Animal Ethics Committee (approval no. IAEC/2010/79). Animals were properly anesthetized before experiments and care were taken in all the animal experiments to minimize the sufferings to the animals. The animal protocols adhered to the guidelines of CPCSEA (Committee for the Purpose of Control and Supervision of Experiments on Animals, registration no. 34/GO/ReBi/S/99CPCSEA, dated 12 March 2015)), Govt. of India.

## Electronic supplementary material

Supplementary Information
