# Supplementary material for: Identification of GXXXXG motif in Chrysophsin-1 and its implication in the design of analogs with cell-selective antimicrobial and anti-endotoxin activities
Source: Sci Rep. 2017 Jun 13;7:3384. doi: 10.1038/s41598-017-03576-1 (PMC5469811; doi:10.1038/s41598-017-03576-1)

Identification of GXXXXG motif in Chrysophsin-1 and its implication in the design of analogs with cell-selective antimicrobial and anti-endotoxin activities

Amit Kumar Tripathi[1], Tripti Kumari[1], Munesh Kumar Harioudh[1], Pranjal Kumar Yadav[2], Manoj Kathuria[3], P.K. Shukla[2], Kalyan Mitra[3], Jimut Kanti Ghosh*[1].

Supporting Information

Figure S1: HPLC traces of Chrysophsin-1 and its proline-substituted analogs

Chrysophsin-1

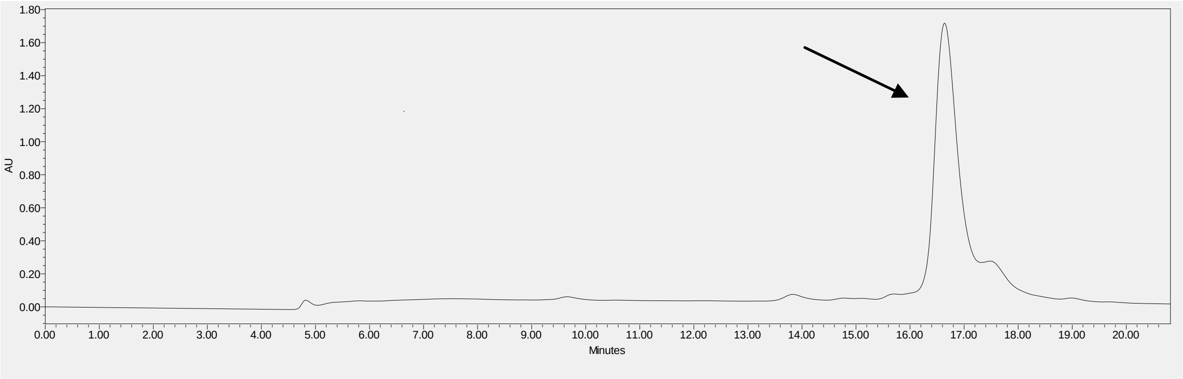

G8P-Chr-1

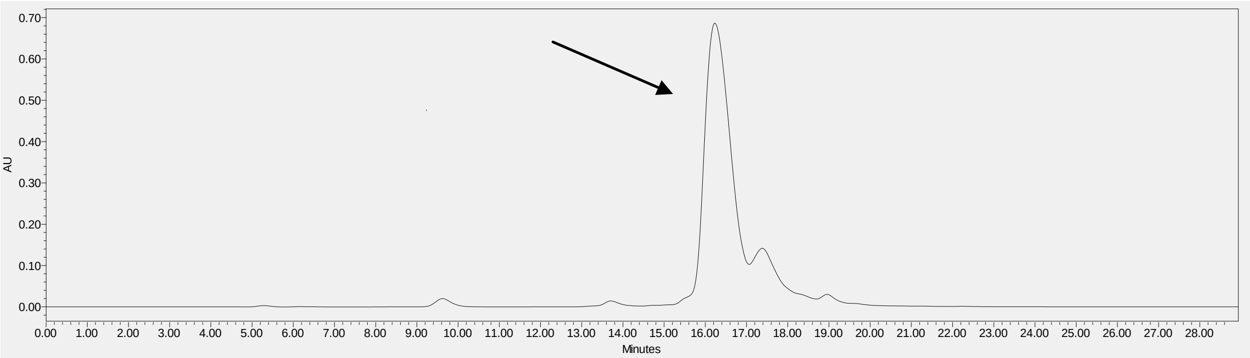

G13P-Chr-1

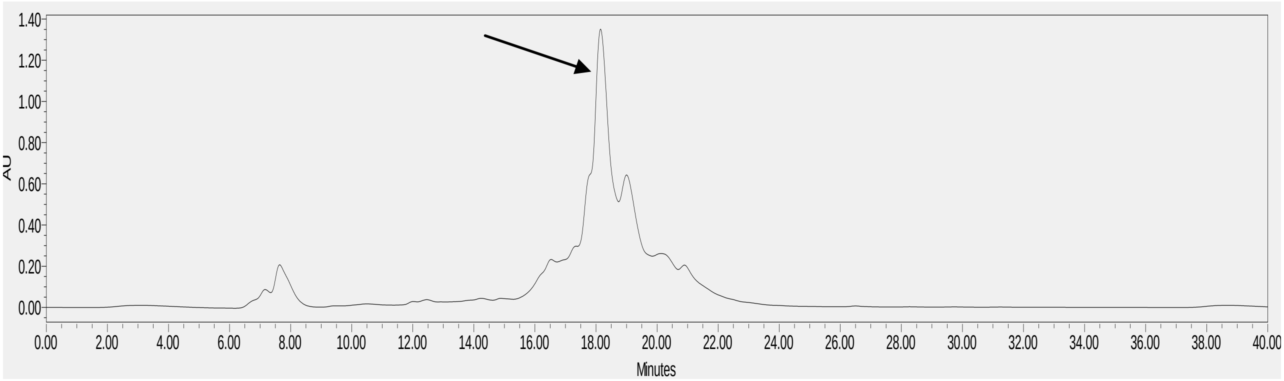

G18P-Chr-1

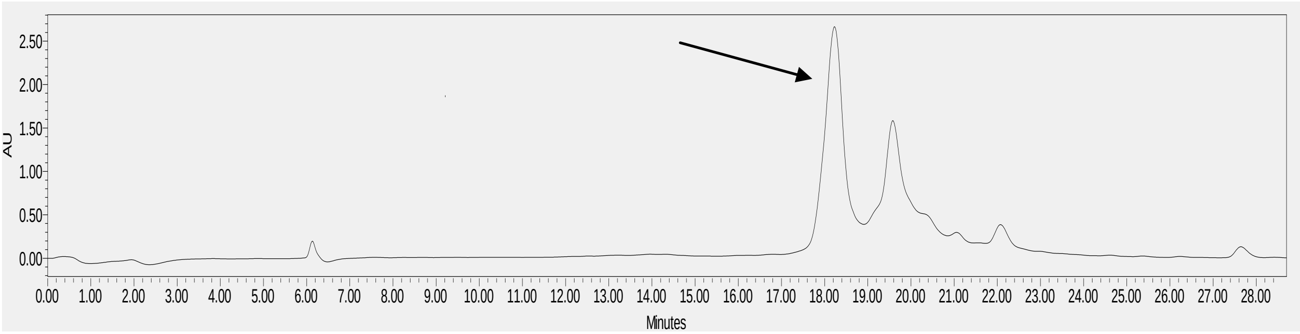

G13,18P-Chr-1

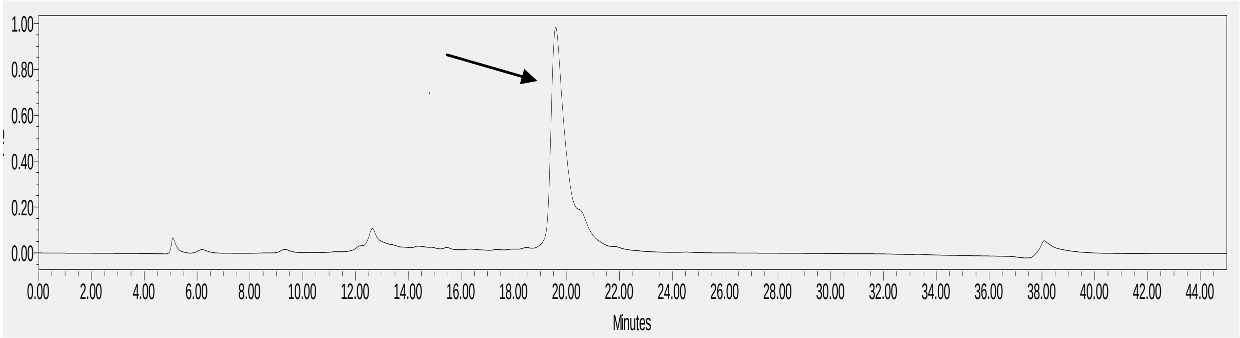

Table S1: Chrysophsin-1-analogs designed by substituting glycine residue(s) in its GXXXXG motifs with alanine/valine residue(s).

Sl.No	Peptide	Sequence	Length	Calculated M.W.	Measured M.W.	Znet	%Hydrophobicity
1	Chrysophsin-1	FFGWLIKGAIHAGKAIHGLIHRRRH	25	2892.79	2892.53	+5	48%
2	8A-Chr-1	FFGWLIKAAIHAGKAIHGLIHRRRH	25	2850.71	2850.61	+5	48%
3	13A-Chr-1	FFGWLIKGAIHAAKAIHGLIHRRRH	25	2906.81	2906.95	+5	52%
4	18A-Chr-1	FFGWLIKGAIHAGKAIHALIHRRRH	25	2906.81	2906.90	+5	52%
5	13,18A-Chr-1	FFGWLIKGAIHAAKAIHALIHRRRH	25	2920.83	2920.71	+5	56%
6	8V-Chr-1	FFGWLIKVAIHAGKAIHGLIHRRRH	25	2934.87	2934.74	+5	52%
7	13V-Chr-1	FFGWLIKGAIHAVKAIHGLIHRRRH	25	2934.87	2934.71	+5	52%
8	18V-Chr-1	FFGWLIKGAIHAGKAIHVLIHRRRH	25	2934.87	2934.78	+5	52%
9	13,18V-Chr-1	FFGWLIKGAIHAVKAIHVLIHRRRH	25	2976.95	2976.75	+5	56%

Figure S2: Hemolytic assay of peptides in Table S1

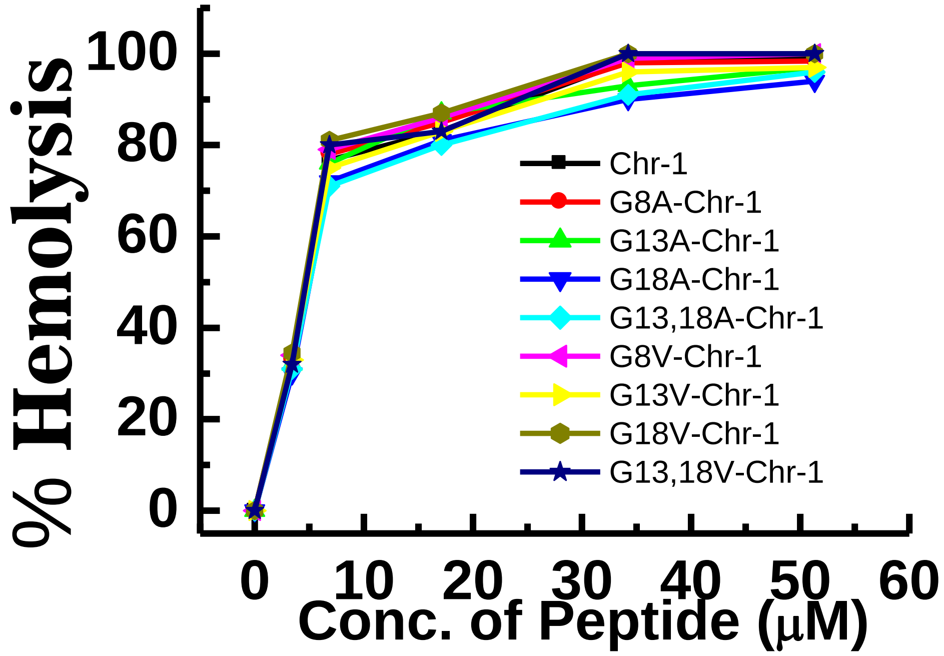

Figure S3: Representative fluorescence profiles for different peptide-induced depolarization of PE/PG [7:3 (w/w)] vesicles at 8 μM concentration for each of the peptides. B, shows the corresponding percent fluorescence recovery induced by these peptides in PE/PG (7:3, w/w) lipid vesicles (lipid concentration ~250 μM).

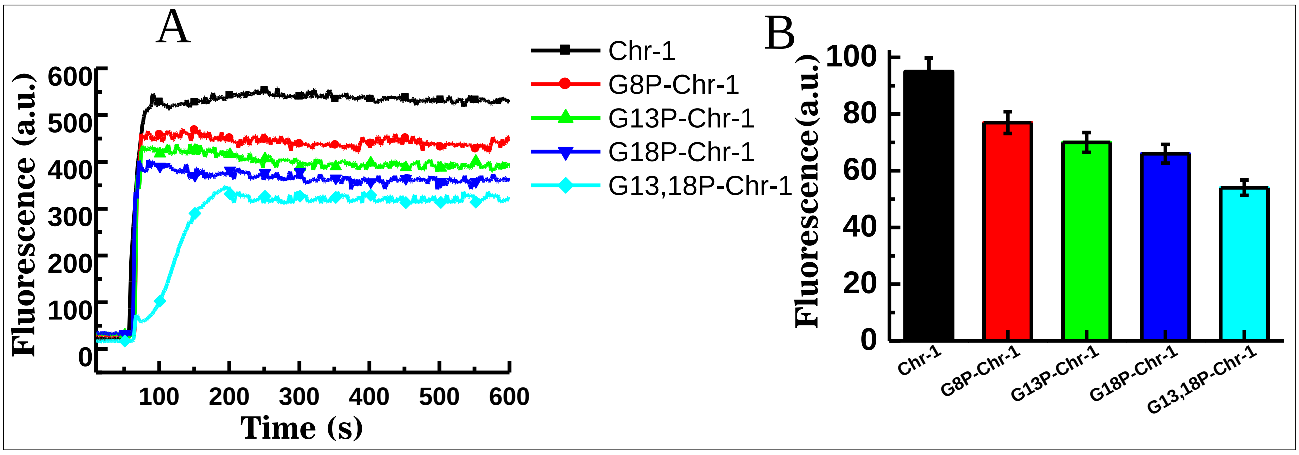

Table S2: Antimicrobial peptides with GXXXXG motif in Antimicrobial peptide database.

Sl.No.	Name/Class	Sequence	ADP ID	Source
1	Epinecidin-1	**GFIFHIIKGLFHAGKMIHGLV**	AP01328	*Epinephelus coioides*
2	moroPC-NH2	**FFGHLFRGIINVGKHIHGLLSG**	AP02786	*Parachaenichthys charcoti*
3	HKPLP (K. kuda pleurocidin-like peptide, fish, animals)	**FLGLIFHGLVHAGKLIHGLIHRNRG**	AP02038	*Hippocampus kuda*
4	Chionodracine (cnd, piscidin-like, fish, animals	**FFGHLYRGITSVVKHVHGLLSG**	AP02159	*Chionodraco hamatus*
5	NRC-19	**FLGLLFHGVHHVGKWIHGLIHGHH**	AP00792	*Hippoglossus hippoglossus* L
6	Of-Pis1	**FLGMLLHGVGHAIHGLIHGKQNVE**	AP02631	*Oplegnathus fasciatus*
7	Piscidin-1	**FFHHIFRGIVHVGKTIHRLVTG**	AP00473	Morone saxatilis

Figure S4: CD spectra of Chrysophsin-1 and its alanine/valine substituted analogs in PC/Chol lipid vesicles respectively at 25 µM concentration of each peptide. Lipid Conc.: ~ 250 µM

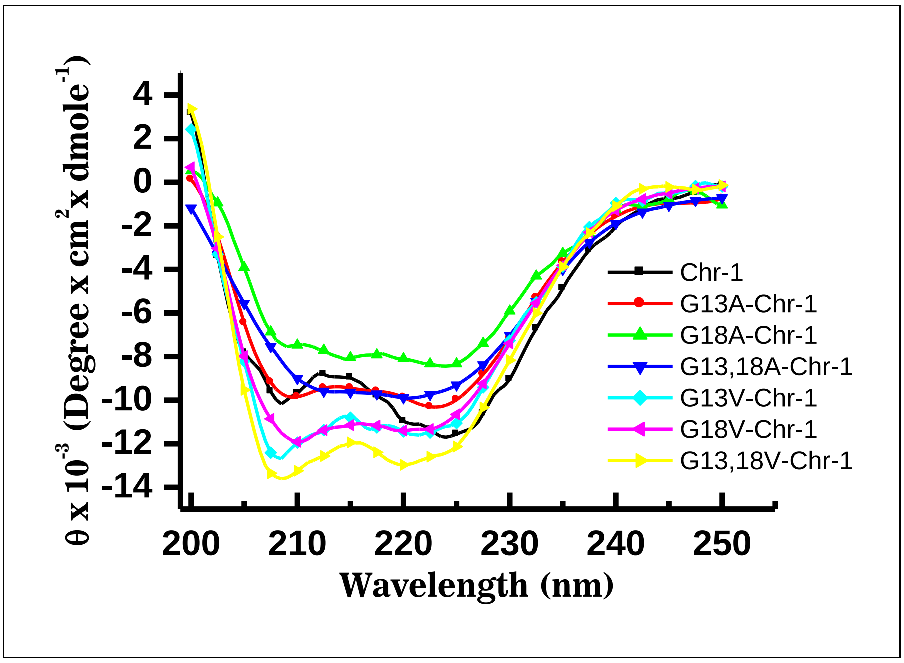

Figure S5: Schiffer–Edmunson helical wheel diagram demonstrating amphipathic α-helical conformation of chrysophsin-1and its proline substituted analogs

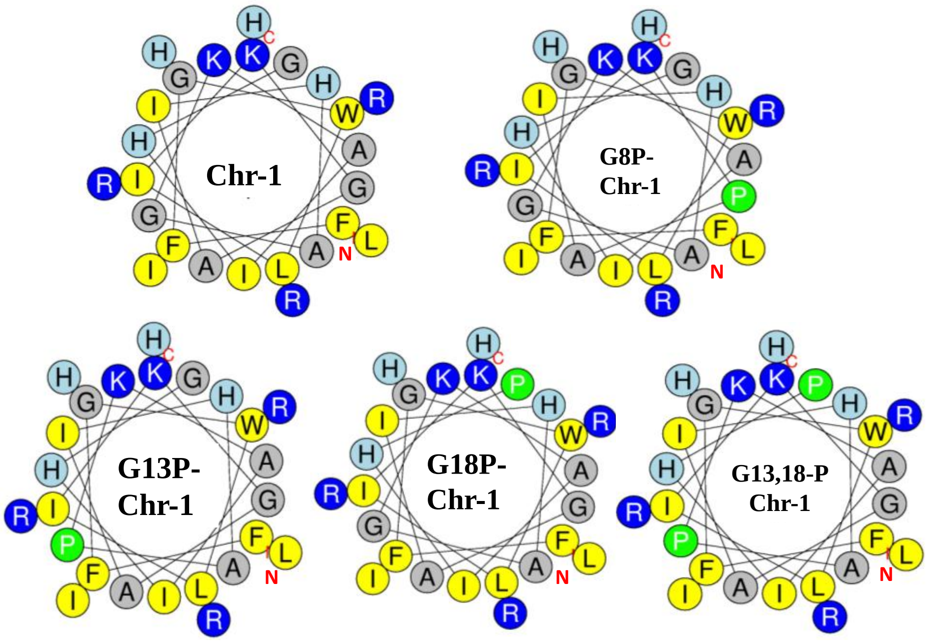

Figure S6: Nitric oxide (N.O.) and TNF-α inhibition in BMDMs by Chrysophsin-1 and its analogs

Quantification of Nitrite Production in LPS-stimulated Bone Marrow Derived macrophages (BMDMs) Cells. Nitrite accumulation in culture media is a function of NO production inside the cell in response to any pro-inflammatory stimuli such as LPS. BMDMs cells were plated at a density of 5 X 10^5 cells/ml in 24-well culture plates and stimulated with LPS(1 μgml^{-1}) from *E. coli* O111:B4 (Sigma) in the presence or absence of peptides for 24 h. Isolated supernatant fractions were mixed with an equal volume of Griess reagent and incubated at room temperature for 10 min. The absorbance was taken at 548 nm to estimate the nitrite production and then quantified by reference to a standard curve generated with $NaNO_2$.

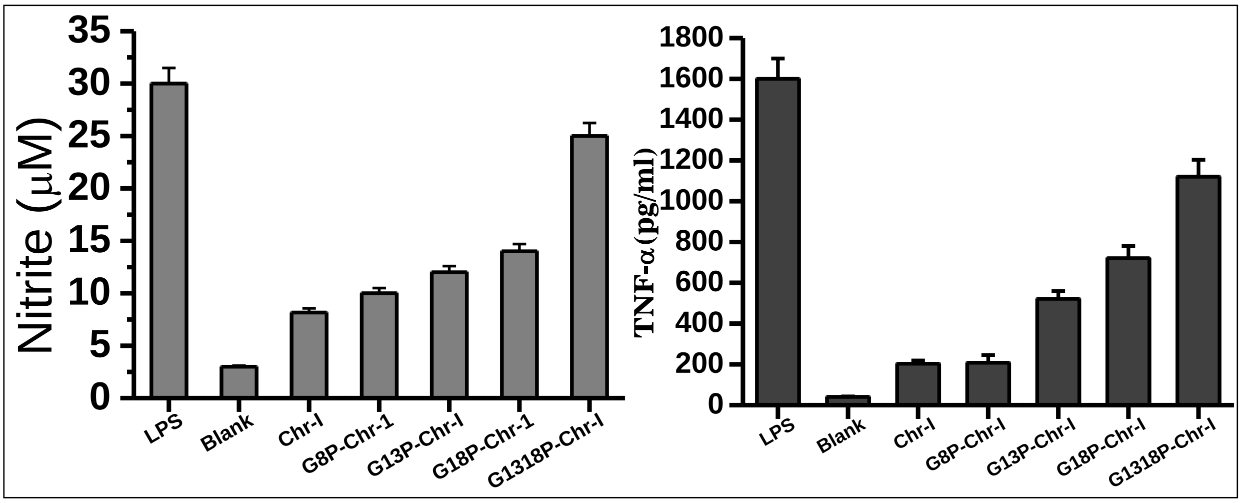

Figure S7: CD experiments performed in presence of LPS. Panel A, CD spectra of peptides in PBS (pH-7.4).Panel B, CD spectra of peptides (25 μM) at LPS 40 μgml^{-1}. Panel C, enhancement of percent helicity of peptides with increasing concentration of LPS . Peptide concentration (25 μM).

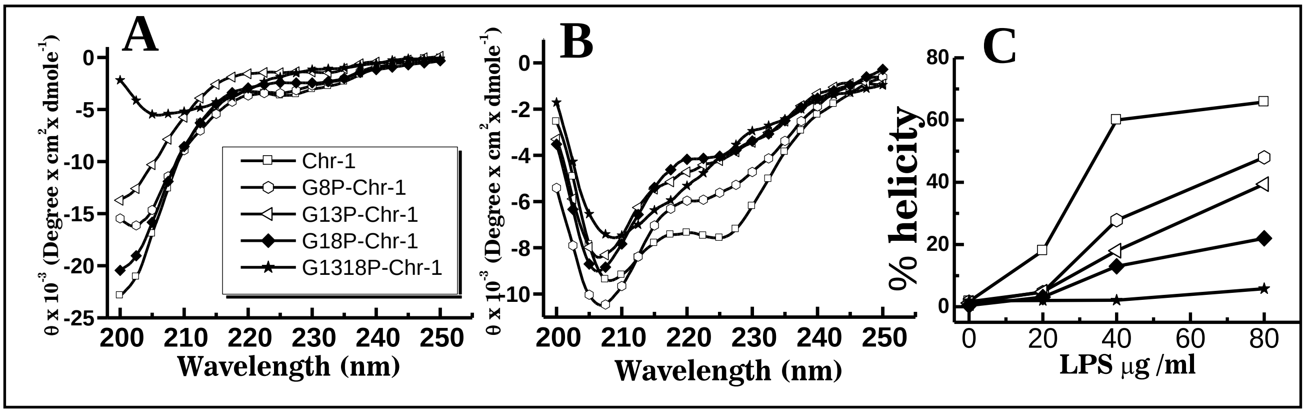

Supplement: Supplementary file 1 — Supplementary Information [file 41598_2017_3576_MOESM1_ESM.pdf]
